# Effectiveness of Traditional Strength vs. Power Training on Muscle Strength, Power and Speed with Youth: A Systematic Review and Meta-Analysis

**DOI:** 10.3389/fphys.2017.00423

**Published:** 2017-06-30

**Authors:** David G. Behm, James D. Young, Joseph H. D. Whitten, Jonathan C. Reid, Patrick J. Quigley, Jonathan Low, Yimeng Li, Camila D. Lima, Daniel D. Hodgson, Anis Chaouachi, Olaf Prieske, Urs Granacher

**Affiliations:** ^1^School of Human Kinetics and Recreation, Memorial University of NewfoundlandSt. John's, NL, Canada; ^2^Tunisian Research Laboratory “Sport Performance Optimisation”, National Center of Medicine and Science in SportsTunis, Tunisia; ^3^Sports Performance Research Institute New Zealand, Auckland University of TechnologyAuckland, New Zealand; ^4^Division of Training and Movement Sciences, Research Focus Cognition Sciences, University of PotsdamPotsdam, Germany

**Keywords:** children, boys, girls, plyometric training, resistance training

## Abstract

Numerous national associations and multiple reviews have documented the safety and efficacy of strength training for children and adolescents. The literature highlights the significant training-induced increases in strength associated with youth strength training. However, the effectiveness of youth strength training programs to improve power measures is not as clear. This discrepancy may be related to training and testing specificity. Most prior youth strength training programs emphasized lower intensity resistance with relatively slow movements. Since power activities typically involve higher intensity, explosive-like contractions with higher angular velocities (e.g., plyometrics), there is a conflict between the training medium and testing measures. This meta-analysis compared strength (e.g., training with resistance or body mass) and power training programs (e.g., plyometric training) on proxies of muscle strength, power, and speed. A systematic literature search using a Boolean Search Strategy was conducted in the electronic databases PubMed, SPORT Discus, Web of Science, and Google Scholar and revealed 652 hits. After perusal of title, abstract, and full text, 107 studies were eligible for inclusion in this systematic review and meta-analysis. The meta-analysis showed small to moderate magnitude changes for training specificity with jump measures. In other words, power training was more effective than strength training for improving youth jump height. For sprint measures, strength training was more effective than power training with youth. Furthermore, strength training exhibited consistently large magnitude changes to lower body strength measures, which contrasted with the generally trivial, small and moderate magnitude training improvements of power training upon lower body strength, sprint and jump measures, respectively. Maturity related inadequacies in eccentric strength and balance might influence the lack of training specificity with the unilateral landings and propulsions associated with sprinting. Based on this meta-analysis, strength training should be incorporated prior to power training in order to establish an adequate foundation of strength for power training activities.

## Introduction

In contrast to the prior myths of health concerns regarding resistance training (RT) for children (Rians et al., 1987; Blimkie, 1992, 1993; Faigenbaum and Kang, 2005), the contemporary research emphasizes the beneficial effect of youth RT for health, strength, and athletic performance (Sale, 1989; Webb, 1990; Faigenbaum et al., 1996, 2009; Falk and Tenenbaum, 1996; Payne et al., 1997; Golan et al., 1998; Hass et al., 2001; McNeely and Armstrong, 2002; Falk and Eliakim, 2003; American College of Sports Medicine, 2006; Faigenbaum, 2006; Malina, 2006; Behm et al., 2008; Granacher et al., 2016). With a properly implemented youth RT program, muscular strength and endurance can increase significantly beyond normal growth and maturation (Pfeiffer and Francis, 1986; Weltman et al., 1986; Sailors and Berg, 1987; Blimkie, 1989; Ramsay et al., 1990; Faigenbaum et al., 1996, 1999, 2001, 2002). Falk and Tenenbaum (1996) conducted a meta-analysis and reported RT-induced strength increases of 13–30% in pre-adolescent children following RT programs of 8–20 weeks. The Canadian Society for Exercise Physiology (CSEP) position stand (Behm et al., 2008) indicated that the literature provided a clear positive effect for improving muscle strength. In contrast, there were far fewer RT studies that measured power capacities, which only provided small effects for adolescents and unclear effects of RT on improving power for children (Weltman et al., 1986; Faigenbaum et al., 1993, 2002, 2007b, 1996; Lillegard et al., 1997; Christou et al., 2006; Granacher et al., 2016).

The small or unclear effects of traditional strength/RT on measures of power in children in the Behm et al. (2008) review could be attributed to the few studies published up to that year that monitored proxies of power. The recent Granacher et al. (2016) review cited only three studies with girls as participants compared to 27 studies with boys but still reported small to barely moderate effects of RT on muscular power. Other factors contributing to smaller effects of traditional strength/RT on measures of power in children could be the lack of training mode specificity (Sale and MacDougall, 1981; Behm and Sale, 1993; Behm, 1995) or perhaps maturation-related physiological limitations upon power training adaptations in children. The typical strength RT protocol for children involves training 2–3 times per week (Malina, 2006), with moderate loads (e.g., 50–60% of 1RM) and higher repetitions (e.g., 15–20 reps) (Faigenbaum et al., 1996, 2009; Lillegard et al., 1997; Christou et al., 2006; Faigenbaum, 2006; Benson et al., 2007; Behm et al., 2008). According to the concept of training specificity, an effective transfer of training adaptations occurs when the training matches the task (e.g., testing, competition) (Sale and MacDougall, 1981; Behm and Sale, 1993; Behm, 1995). Since high power outputs involve explosive contractions with forces exerted at higher velocities, RT programs using low to moderate loads at slower velocities would not match power characteristics. However, recently there are a number of publications that have implemented power training programs (e.g., plyometric training) for children that would adhere to the training specificity principle. Plyometric exercises involve jumping, hopping, and bounding exercises and throws that are performed quickly and explosively (Behm, 1993; Behm et al., 2008; Cappa and Behm, 2011, 2013). With plyometric training adaptations, the neuromuscular system is conditioned to react more rapidly to the stretch-shortening cycle (SSC). Plyometric training can be safe and may improve a child's ability to increase movement speed and power production provided that appropriate training and guidelines are followed (Brown et al., 1986; Diallo et al., 2001; Matavulj et al., 2001; Lephart et al., 2005; Marginson et al., 2005; Kotzamanidis, 2006; Behm et al., 2008). Johnson et al. (2011) published a meta-analysis that only included seven studies that they judged to be of low quality. They suggested that plyometric training had a large positive effect on running, jumping, kicking distance, balance, and agility with children. Hence, further analysis is needed with a greater number of power training studies involving children and/or adolescents.

While many power activities involve shorter duration, higher intensity, explosive type contractions (anaerobic emphasis), children are reported to possess reduced anaerobic capacities (Behm et al., 2008; Murphy et al., 2014) with a lower reliance on glycolysis (Ratel et al., 2006, 2015), and lower power outputs (Falk and Dotan, 2006) compared to adults. In the recently published scoping review (Granacher et al., 2016), Granacher and colleagues were able to show small effect sizes following RT on measures of power in child athletes and moderate effect sizes in adolescent athletes. However, these authors looked at general RT effects only and did not differentiate between strength and power training programs. Moreover, only studies conducted with youth athletes were analyzed.

Thus, it was the objective of this systematic review and meta-analysis to investigate whether there are different effects following strength vs. power training on measures of muscle strength, power, and speed in trained and untrained children and adolescents. It is hypothesized that in accordance with the concept of training specificity, power training programs will provide more substantial improvements in power and speed measures than traditional strength programs with youth. Furthermore, since trained individuals would have a greater foundation of strength, we expected greater power training related effects in trained compared to untrained youth.

## Methods

### Search strategy and inclusion/exclusion criteria

This review included randomized controlled trials and controlled trials that implemented either traditional strength/resistance training or power training in youth. A literature search was performed by four co-authors separately and independently using PubMed, SPORT Discus, Web of Science, and Google Scholar databases. The topic was systematically searched using a Boolean search strategy with the operators AND, OR, NOT and a combination of the following keywords: (“strength training” OR “resistance training” OR “weight training” OR “power training” OR “plyometric training” OR “complex training” OR “compound training” OR “weight-bearing exercise”) AND (child OR children OR adolescent OR adolescents OR youth OR puberty OR prepuberal^*^ OR kids OR kid OR teen^*^ OR girl^*^ OR boy OR boys) NOT (patient OR patients OR adults OR adult OR man OR men OR woman OR women). All references from the selected articles were also crosschecked manually by the authors to identify relevant studies that might have been missed in the systematic search and to eliminate duplicates.

### Inclusion criteria (study selection)

**Figure 1:**
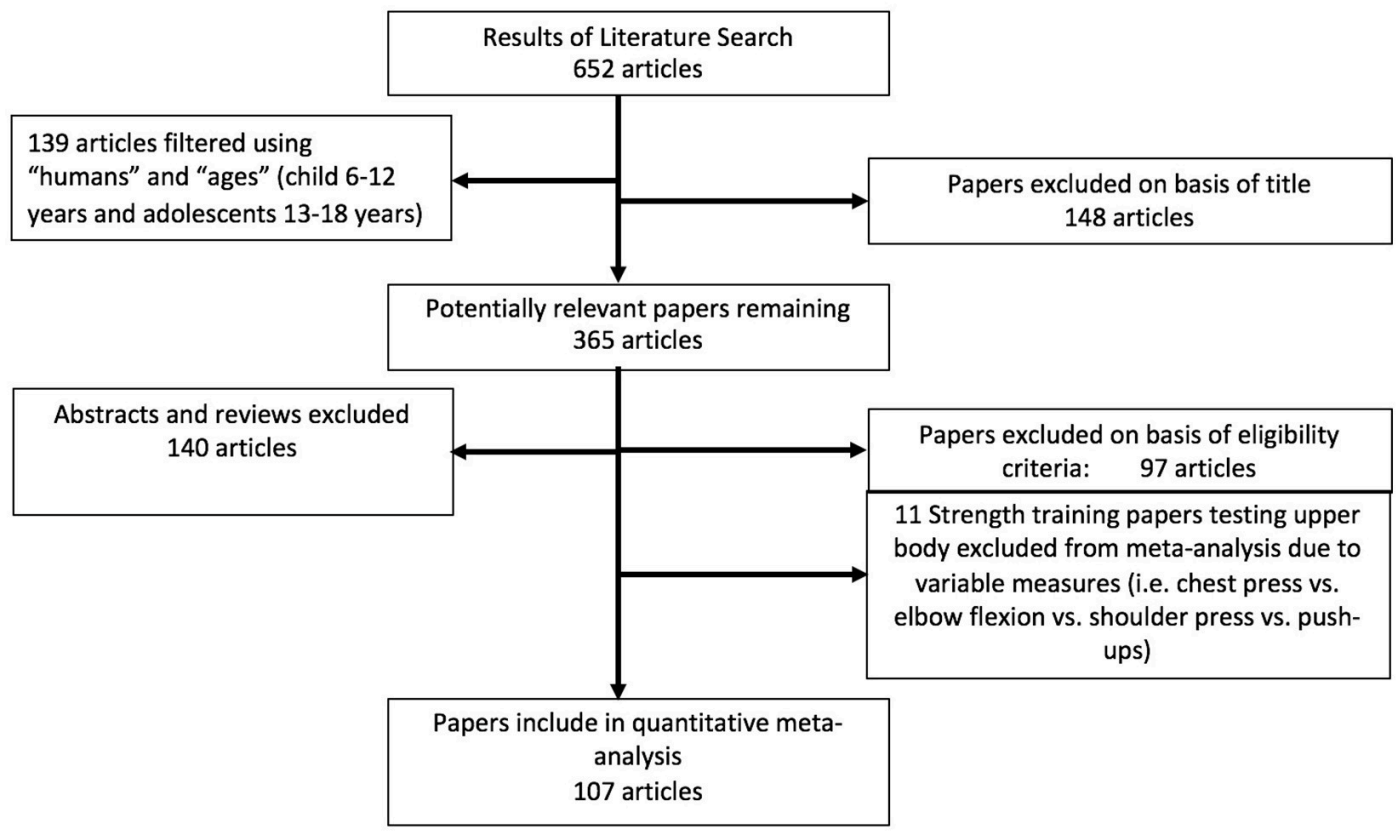
Flow chart illustrating the different phases of the search and study selection.

Studies investigating traditional strength/resistance training or power training in youth were included in the review if they fulfilled the following selection criteria: the study (1) was a randomized controlled trial or a controlled trial; (2) measured pre- and post-training strength [e.g., maximal loads (i.e., 1 repetition maximum: 1RM) or forces with squats, leg extension or flexion, isokinetic maximal measures], power-related [e.g., countermovement jump (CMJ), horizontal or standing long jump (SLJ)] or speed-related (e.g., 10-m sprint time) dependent variables; (3) training duration was greater than 4 weeks; (4) used healthy, untrained (i.e., physical education classes and/or no specific sport) or trained (i.e., youth athletes from different sports) youth participants under the age of 18 years; (5) was written in English and published prior to January 2017; and (6) was published in a peer-reviewed journal (abstracts and unpublished studies were excluded). Studies were excluded if precise means and standard deviations, or effect sizes were not available or if the training study combined both strength and power exercises. Our initial search resulted in 652 applicable studies (see flow chart: Figure 1).

### Statistical analyses

For statistical analyses, within-subject standardized mean differences of the each intervention group were calculated [SMD = (mean post-value intervention group—mean pre-value intervention group)/pooled standard deviation]. Subsequently, SMDs were adjusted for the respective sample size by using the term (1-(3/(4N-9))) (Hedges, 1985). Meta-analytic comparisons were computed using Review Manager software V.5.3.4 (Copenhagen: The Nordic Cochrane Centre, The Cochrane Collaboration, 2008). Included studies were weighted according to the magnitude of the respective standard error using a random-effects model. A random effect model was used because the relative weight assigned to each of the studies has less impact on computed combined effect size. In other words, in the fixed effect model, one or two studies with relatively high weight can shift the combined effect size and associated confidence intervals in one particular direction, whereas in a random effect model this issue is moderated.

Further, we used Review Manager for subgroup analyses: computing a weight for each subgroup (e.g., trained vs. untrained), aggregating SMD values of specific subgroups, and comparing subgroup effect sizes with respect to differences in intervention effects across subgroups. To improve readability, we reported positive SMDs if superiority of post values compared with pre-values was found. Heterogeneity was assessed using I^2^ and χ^2^ statistics. SMDs were calculated to evaluate the magnitude of the difference between traditional resistance and plyometric training according to the criterion of 0.80 large; 0.50 medium and 0.20 small (Cohen, 1988).

## Results

### Training program prescriptions

The descriptive statistics for the strength and power training program prescriptions are illustrated in Table 1. There were 28.5% more strength training studies within the literature review likely due to the fact that power training experiments for children began more recently (power: 1999 vs. strength: 1986 with one pediatric strength study published in 1958). Strength training studies on average had younger participants (~12 vs. 13 years), 45.2% longer duration training programs (~8 vs. 12 weeks) and implemented approximately 1 less exercise per training session. There were substantially more untrained or physical education student participants in the strength studies (i.e., strength studies with physical education and untrained: 31 vs. power studies with physical education and untrained: 6 with soccer athletes used most often (strength: 9 studies and power: 20 studies). Details of all studies in the review are depicted in Tables 2A,B.

**Table 1:** Training participants and program characteristics.

**No. of Studies**	**No. of studies with all male subjects**	**No. of studies with all female subjects**	**No. of studies with male and female subjects**	**Age (years)**	**Training frequency (sessions per week)**	**Training Weeks**	**Sets**	**No. of Exerc**.	**Reps**
Strength 63 (1958, and 1986–2016)	32	1	30	12.37 ± 0.73	2.2 ± 0.52	12.45 ± 14.04	2.76 ± 1.16	6.15 ± 2.94	9.83 ± 4.08
Power 52 (1999–2016)	38	11	3	13.5 ± 0.86	2.27 ± 0.58	8.57 ± 4.34	2.15 ± 1.81	7.69 ± 4.94	9.94 ± 7.91

*reps, repetitions; Exerc, exercises. Values provided in first four columns are sums, whereas the last six columns are means and standard deviations*.

***Number of studies:** Strength participants: Physical Education: 15 studies, Untrained: 16 studies*.

*Sports: Soccer: 9 studies, Rugby: 4, Gymnasts: 2, Basketball, 2, Baseball: 2, Football: 2, Swimming, Handball, American Rowing, Judo, Wrestling, and assorted other sports or trained states*.

*Power participants: Physical Education: 3 studies, Untrained: 3 studies*.

*Sports: Soccer: 20 studies, Rugby: 4, Gymnasts: 2, Basketball, 6, Swimming: 2, Volleyball: 2, Baseball, American Football, Handball, Rowing, Judo, Wrestling, Rowing, Track, Field hockey, Tennis, and assorted other sports or trained states*.

**Table 2A:** Strength type resistance training program descriptions.

**Article**	**Tr**	**Sex**	**Age**	***N***	**Freq**	**Wks**	**Sets**	**Ex**	**Reps**	**Int**	**Strength**	**Pre**	***SD***	**Post**	***SD***	**%Δ**	**Power**	**Pre**	***SD***	**Post**	***SD***	**%Δ**
Assuncao et al., 2016	U	MF	Low rep.: 13.8 ± 0.9	17	2	9	2	8	4–6	4–6 RM	Low rep.:											
			High rep.: 13.7 ± 0.7	16	2	9	2	8	12–15	12–15 RM	1 RM chest press	Effect sizes only										
											1 RM squat	Effect sizes only										
											High rep.:											
											1 RM chest press	Effect sizes only										
											1 RM squat	Effect sizes only										
Benson et al., 2007	PE	MF	12.3 ± 1.3	32	2	8	2	11	8	RPE 15–18	Bench press	29.6	8.2	41.1	9.5	38.9						
											Bench press/kg	0.5	0.1	0.7	0.2	40.0						
											Leg press	109.2	39.1	152.1	43.4	39.3						
											Leg press/kg	1.9	0.4	2.6	0.7	36.8						
Blimkie, 1989		M	10.4 ± 0.8	14	3	10	3–5	6	10–12		EF MVIC 100°	14.7	5.3	18.0	4.8	22.4						
Channell and Barfield, 2008	T	M	15.9 ± 1.2	21	3	8	3–5	2–4	3–20	60–100%	Squat	144.0	41.6	161.6	29.3	12.2	CMJ	0.6	0.7	0.6	0.4	3.4
											Power clean	72.6	17.8	84.3	15.6	16.1						
											Squat	132.6	30.9	160.9	26.0	21.3	CMJ	0.5	0.9	0.5	0.9	2.1
											Power clean	69.2	17.8	70.1	12.9	1.3						
Chelly et al., 2009	T	M	17.0 ± 0.3	11	2	8	3	1	2-4	80–90%	Squat	105.0	14.0	142.0	15.0	35.2	SJ	0.3	0.0	0.4	0.0	9.4
																	CMJ	0.3	0.0	0.4	0.0	5.9
																	5 Long jump	10.6	0.3	11.1	0.2	4.7
																	5 m Velocity m/s	3.5	0.2	3.8	0.1	7.1
																	40 m Max velocity m/s	7.8	0.5	8.8	0.4	11.9
Christou et al., 2006	T	M	13.8 ± 0.4	9	2	16	2–3	10	8–15	55–80%	Leg press	102.8	2.5	163.9	7.4	59.4	SJ	0.3	0.0	0.3	0.0	12.0
											Bench press	36.0	1.6	55.0	3.1	52.8	CMJ	0.3	0.0	0.4	0.0	20.0
																	10 m Sprint	2.2	0.1	2.1	0.0	3.2
																	30 m Sprint	5.1	0.2	4.9	0.1	2.6
Contreras et al., 2017	T	M	15.5 ± 1.2	13	2	12	1	4	6–12	6–12 RM	Front squat	77.6	12.4	83.1	13.8	7.1	CMJ	0.6	0.1	0.6	0.8	3.6
	T		Hip thrust														Long jump	2.3	0.2	2.4	0.2	16.3
																	10 m Sprint	1.8	0.1	1.7	0.1	1.1
																	20 m Sprint	3.1	0.1	3.1	0.1	1.9
			15.5 ± 0.7	11							Front squat	75.0	10.5	84.6	10.0	12.9	CMJ	0.5	0.1	0.6	0.1	7.7
			Front squat														Long jump	2.3	0.2	2.3	0.3	1.8
																	10 m Sprint	1.8	0.1	1.8	0.1	0.6
																	20 m Sprint	3.2	0.1	3.1	0.2	–0.6
Coskun and Sahin, 2014	U	MF		18	2	6	2	8	10–12	10 RM	Leg press	27.1	8.6	42.7	12.6	57.6						
Dalamitros et al., 2015	T	M	14.8 ± 0.5	11	2	24		4			KE PT 60 R	196.6	61.6	209.8	45.8	6.7						
											KE PT 60 L	188.5	47.0	206.4	44.2	9.5						
											KF PT 60 R	102.8	28.7	108.5	29.0	5.5						
											KF PT 60 L	100.7	28.6	107.5	29.7	6.8						
Dorgo et al., 2009	PE	MF	16.0 ± 1.2	63	3	18	12–28		10–14		Push up	9.7	1.1	12.6	1.1	29.9						
											Pull up	7.6	0.9	12.1	0.9	59.2						
dos Santos Cunha et al., 2015	U	M	10.4 ± 0.5		3	12	3	7	6–15	60–80%	EF 1 RM kg	6.4	0.8	10.6	0.9	65.6						
											EF 1 RM kg/FFM	2.6	0.3	4.3	0.6	65.4						
											EF Isok 30	4.9	1.2	6.1	1.3	24.5						
											EF Isok 90	4.4	0.8	5.2	0.8	18.2						
											EF Isom 45	4.4	1.4	6.0	1.1	36.4						
											EF Isom 90	7.7	1.9	9.3	1.8	20.8						
											KE 1 RM kg	10.8	1.6	18.7	2.0	73.1						
											KE 1 RM kg/FFM	1.1	0.1	2.0	0.1	81.8						
											KE Isok 30	7.6	1.1	9.6	1.3	26.3						
											KE Isok 90	6.5	0.5	8.3	0.7	27.7						
											KE Isom 45	8.5	1.6	9.5	1.2	11.8						
											KE Isom 90	9.8	1.5	11.8	1.7	20.4						
											KE PT 60 R	196.6	61.6	209.8	45.8	6.7						
											KE PT 60 L	188.5	47.0	206.4	44.2	9.5						
											KF PT 60 R	102.8	28.7	108.5	29.0	5.5						
											KF PT 60 L	100.7	28.6	107.5	29.7	6.8						
											Overhead press 10 RM	7.5	2.5	14.1	3.1	87.0						
Faigenbaum et al., 1996	U	MF	10.8 ± 0.4	15	2	8	2–3	5	6	8 RM	Leg extension 6 RM	18.0	1.8	28.0	4.6	55.6	CMJ	23.5	1.4	24.9	1.5	6.0
											Chest press 6 RM	21.8	2.2	30.1	4.6	38.1						
Faigenbaum et al., 1999	U	MF	7.8 ± 1.4	15	2	8	1	11	6–8	Fail	Chest press	24.5	5.9	25.8	6.4	5.3						
											Leg extension	18.4	7.0	24.1	7.6	31.0						
			8.5 ± 1.6	16					13–15	Fail	Chest press	25.7	9.1	29.9	9.7	16.3						
											Leg extension	19.3	9.0	27.2	10.9	40.9						
Faigenbaum et al., 2001	U	MF	8.1 ± 1.6	44	2	8	1	1	6–8		Heavy load chest press	24.5	5.9	25.8	6.4	5.3						
									13-15		Moderate load chest press	25.7	9.1	29.9	9.7	16.3						
											Heavy med ball chest press	23.8	4.3	27.8	4.1	16.8						
											Med ball chest press	24.1	3.9	25.8	3.8	7.1						
Faigenbaum et al., 2002	U	MF	9.7 ± 1.4	20	2	8	1	12	10–15	10–15 RM	Chest press	21.7	7.0	24.2	7.7	11.5	Long jump	129.5	5.6	139.5	15.6	7.7
											Leg press	56.9	24.0	71.1	27.5	25.0	CMJ	22.8	3.9	24.9	4.5	9.2
											Grip strength	35.8	6.9	38.2	7.4	6.7						
Faigenbaum et al., 2007a	PE	MF	13.9 ± 0.4	22	2	9	3	9	7	12–15 RM	Squat 10 RM	56.9	15.0	67.6	15.4	18.8	Med ball throw	356.5	55.1	368.3	59.1	3.3
											Bench press 10 RM	41.1	9.4	47.4	10.5	15.3	CMJ	48.9	6.6	51.1	8.6	4.5
Faigenbaum et al., 2014	PE	MF	7.5 ± 0.3	21	2	8	2	5	7–10		Pull up	7.6	0.9	10.3	0.9	35.5	Long jump	111.9	11.2	116.4	11.6	4.0
											Push up	1.9		2.5		31.6						
Faigenbaum et al., 2015	PE	MF	9.5 ± 0.3	20	2	8	1	13	30–45 Sec		Push up	11.5	0.9	15.9	1.8	38.3	Long jump	121.2	5.4	130.2	6.3	7.4
Faigenbaum et al., 2007b	T	M	13.6 ± 0.7	14	2	6	3	6	10–12	12 RM							CMJ	48.2	10.7	49.6	10.1	2.9
																	Long jump	190.0	23.1	192.2	25.4	1.2
																	Ball toss	321.7	58.0	339.4	85.2	5.5
Falk and Mor, 1996		MF	6.4 ± 0.4	14	2	12	3	4	15	15 RM							Long jump	101.0	13.0	115.0	18.0	13.9
																	Ball put	233.0	28.0	244.0	43.0	4.7
Ferrete et al., 2014	T	MF	9.3 ± 0.3	11	2	26	2–4	6	6–10								CMJ	22.3	0.7	23.8	4.3	6.7
Flanagan et al., 2002	T	MF	8.8 ± 0.5	14	2	11	1–3	8	10–15								Med ball (resisted)	321.8	29.9	336.0	26.0	4.0
	T		8.6 ± 0.5	24	2	11	Var	5	Var								Long jump (resisted)	134.3	32.8	148.7	25.9	9.0
																	Med ball (BW)	234.3	34.5	267.9	39.2	12.0
																	Long jump (BW)	138.5	21.8	144.8	16.4	4.0
Funato et al., 1986	PE	MF	11.0 ± 0.3	20	3	12	2	1	3	100% MVIC	EF MVIC					5.7						
											EE MVIC					17.5						
Gonzalez-Badillo et al., 2015	T	M	U16: 14.9 ± 0.3	17	2	26	2–4	7	5–10 and 20 m	40-105%							CMJ (U16)	35.4	3.9	39.1	4.9	10.5
			U18: 17.8 ± 0.4	16	2	26	2–4	7	5–10 and 20 m	40–105%							CMJ (U18)	38.4	3.0	41.3	4.5	7.6
Gorostiaga et al., 1999	T	M	15.1 ± 0.7	9	2	6	4	5		40–90%	KE force	208.0	29.1	235.8	41.1	13.4	Throwing velocity	71.7	6.7	74.0	7.0	3.2
																	SJ	32.2	3.2	33.3	3.3	3.4
	T		15.1 ± 0.5								KF force	100.0	12.2	109.0	15.4	9.0	CMJ	34.1	3.1	35.2	3.6	3.2
Granacher et al., 2010	PE	MF	8.6 ± 0.5	17	2	10	3	5	10–12	70–80%	Isok KE 60	40.1	8.6	47.8	8.7	19.2	CMJ	21.5	2.6	22.2	2.7	3.3
											Isok KF 60	32.8	5.2	37.1	7.1	13.1						
											Isok KE 180	33.1	5.4	38.3	6.6	15.7						
											Isok KF 180	28.7	3.6	32.1	4.2	11.8						
Granacher et al., 2011b	PE	MF	16.7 ± 0.6	14	2	8	4	7	10	30–40%	KE MVIC	1501	404	1632	304	8.7	CMJ	27.4	4.5	29.5	4.9	7.7
										Ballistic												
Granacher et al., 2011a	PE	MF	8.6 ± 0.5	17	2	10	3	7	10–12	70–80%	Iso KE 60	40.1	8.6	47.8	8.7	19.2	CMJ	21.5	2.6	22.2	2.7	3.3
											Isok KF 60	32.8	5.2	37.1	7.1	13.1						
											Isok KE 180	33.1	5.4	38.3	6.6	15.7						
Granacher et al., 2014	T	MF	13.7 ± 0.6	13	2	6	3	3	40–50 s or 20–25		CSTS ventral TMS	65.5	37.0	74.7	39.3	14.0	CSTS long jump	187.6	47.4	189.6	39.0	1.1
			13.8 ± 0.9	14	2	6	3	3	40–50 s or 20–25		CSTS dorsal TMS	152.2	98.0	214.8	32.8	41.1						
											CSTS lateral right TMS	46.9	18.9	51.1	18.3	9.0						
											CSTS lateral left TMS	46.5	20.8	51.4	18.7	10.5	CSTU long jump	201.1	20.0	207.1	18.8	3.0
											CSTU ventral TMS	67.9	34.1	83.1	28.7	22.4						
											CSTU dorsal TMS	129.9	55.0	173.3	20.4	33.4						
											CSTU lateral right TMS	46.7	12.1	50.4	14.7	7.9						
											CSTU lateral left TMS	201.1	10.3	51.4	10.6	8.0						
Hammami et al., 2016b	T	M	BPT: 12.7 ± 0.3	12	3	8	2	5	8–15	Max	Isok KF 180	28.7	3.6	23.1	4.2	−19.5	BPT CMJ	25.5	4.0	29.2	2.9	14.5
			PBT: 12.5 ± 0.3	12	3	8	2	5	8–15	Max							PBT CMJ	24.7	2.4	26.8	1.8	8.5
																	BPT long jump	186.0	15.9	220.7	10.3	18.7
																	PBT long jump	177.1	11.6	206.8	13.9	16.8
																	Bench press throw	87.9	16.9	97.5	21.2	10.9
Harries et al., 2016	T	M	16.8 ± 1.0	8	2	6	1–6	10	3–10	60–90%	Squat	127.9	26.4	171.2	41.2	33.9						
											Bench press	87.9	16.9	97.5	21.2	10.9						
Hettinger, 1958	NR	MF	<2.9: 12.6 ± 3.8	9	1–2	8–23	1	2	1	Max	Lower arm flexors (boys maturity <2.9)	10.9	1.7	13.0	2.1	19.3						
			>3.0: 12.7 ± 2.3	15							Lower arm flexors (girls maturity <2.9)	9.4	1.3	10.7	1.5	13.8						
											Lower arm extensors (boys maturity <2.9)	7.7	1.0	10.8	2.4	40.3						
											Lower arm extensors (girls maturity <2.9)	6.3	1.3	8.7	1.3	38.1						
											Lower Arm Flexors (Boys Maturity >3.0)	19.0	3.9	23.6	4.8	24.2						
											Lower arm flexors (girls maturity >3.0)	16.2	3.6	17.3	3.6	6.8						
											Lower arm extensors (boys maturity >3.0)	13.1	1.3	19.0	4.7	45.0						
											Lower arm extensors (girls maturity >3.0)	10.1	2.1	12.6	2.4	24.8						
Ignjatovic et al., 2011	T	M	15.7 ± 0.8	23	2	12	3	9	8–12	8–12 RM	30% Bench press	367.2	65.4	392.4	61.3	6.9						
											40% Bench press	405.3	71.8	432.0	68.1	6.6						
											50% Bench press	455.9	84.5	475.0	77.1	4.2						
											60% Bench press	503.0	86.6	526.8	82.0	4.7						
Kotzamanidis et al., 2005	T	M	17.1 ± 1.1	11	2	9	4		3-8	8,6,3 RM	Half squat	140.5	15.6	154.5	15.7	10.0	Squat jump	25.7	3.1	26.2	3.5	1.9
											Step up	65.5	7.6	76.4	7.1	16.7	40 cm DJ	18.4	5.5	18.9	5.5	2.6
											Leg curl	53.6	6.7	62.3	5.6	16.1	CMJ	27.2	3.4	27.5	3.3	0.9
																	30 m Sprint	4.3	0.2	4.3	0.2	0.5
Lloyd et al., 2016	PE	M	Pre-PHV: 12.7 ± 0.3	10	2	6	3	4	10	10 RM							Pre-PHV squat jump	22.3	4.9	24.8	4.6	11.2
			Post-PHV: 16.3 ± 0.3	10													Post-PHV squat jump	32.4	5.0	34.6	5.1	6.8
																	Pre-PHV 10 m sprint	2.3	0.2	2.2	0.2	4.4
																	Post-PHV 10 m sprint	1.9	0.1	1.8	0.1	5.3
																	Pre-PHV 20 m sprint	3.4	0.3	3.4	0.3	0.0
																	Post-PHV 20 m sprint	2.8	0.2	2.7	0.2	3.6
Lubans et al., 2010	U	MF	15.0 ± 0.7	F: 37	2	8	2	10	8–12	RPE 15–18	Girls (bench press) FW	49.9	13.0	62.0	11.9	24.2						
				E: 41	2	8	2	10	8–12	RPE 15–18	Girls (incline leg press) FW	173.6	47.2	234.3	50.5	35.0						
											Girls (bench press) ET	50.5	15.2	56.5	14.5	11.9						
											Girls (incline leg press) ET	181.4	53.3	283.6	64.3	56.3						
											Boys (bench press) FW	31.2	6.2	36.4	6.7	16.7						
											Boys (incline leg press) FW	144.8	34.2	191.0	51.3	31.9						
											Boys (bench press) ET	31.7	7.2	35.9	7.1	13.2						
											Boys (incline leg press) ET	156.2	20.0	186.2	30.1	19.2						
Moore et al., 2013	T	M	16.0 ± 2.0	14	3	20	3		20	Low	Posterior shoulder endurance test	30.0	14.0	88.0	36.0	193.3						
Moraes et al., 2013	U	M	15.5 ± 0.9	14	3	12	3	9	10–12	10–12 RM	Bench press	40.6	6.1	48.3	7.2	19.0	Long jump	137.1	22.6	139.8	21.5	2.0
											Leg press	231.4	39.3	435.7	37.0	88.3	CMJ	29.4	6.0	30.8	6.0	4.8
Muehlbauer et al., 2012	PE	M	16.8 ± 0.8	6	2	8	4–6	7	10	30–40%	Boys leg press MVIC	1786	319	1912	280	7.0	Boys CMJ	31.1	2.2	33.3	2.9	7.1
		F	16.6 ± 0.5	8							Girls leg press MVIC	1287	329	1639	325	27.3	Girls CMJ	24.6	3.7	26.7	4.1	8.5
Negra et al., 2016	T	M	12.8 ± 0.2	13	2	12	4	1	8–12	40–60%	Squat	102.0	25.2	127.8	15.2	25.3	Long jump	1.7	0.2	1.9	0.2	15.5
																	CMJ	24.1	4.6	29.8	3.4	23.5
Ozmun et al., 1994	U	MF	10.5 ± 0.6	8	3	8	3	1	7–10	10 RM	Isok EF					27.8						
											Isot EF					22.6						
Piazza et al., 2014	T	M	12.0 ± 1.8	19	2	6	3	12	12	12 RM							Squat jump	427.1	35.3	440.1	28.0	3.0
																	CMJ	449.7	34.5	481.3	30.8	7.0
Pikosky et al., 2002			11.0		2	6	1	9	15	15 RM	Chest press	22.7	1.5	25.1	1.7	10.6						
											KE	18.6	2.4	31.1	3.2	67.2						
Pesta et al., 2014	U	M	15.3 ± 1.0	13	3	10	2	3	12		KE MVIC	1245	226	1329	225	6.8	Squat jump	31.5	3.6	35.9	5.9	14.0
																	CMJ	33.5	4.4	37.8	5.8	12.8
Prieske et al., 2016	T	M	16.6 ± 1.1	20	2–3	9	2–3	5	15–20		Trunk flexors MVIC	657	92	681	89	3.7	CMJ	36.0	3.4	35.5	3.2	–1.4
(Stable)											Trunk extensors MVIC	603	98	644	93	6.8	20 m Sprint	3.0	0.1	3.0	0.1	0.3
Prieske et al., 2016	T	M	16.6 ± 1.0	19	2–3	9	2–3	5	15–20		Trunk flexors MVIC	624	99	617	97	–1.1	CMJ	34.0	3.4	34.3	2.7	0.9
(Unstable)											Trunk extensors MVIC	591	67	614	115	3.8	20 m Sprint	3.0	0.1	3.0	0.1	0.7
Ramsay et al., 1990	U	M	9–11	13	3	20	3–5	6	Failure	70–85%	Bench press					34.6						
											Leg press					22.1						
											Isok PT EF					25.8						
											Isok PT KE					21.3						
Rhea et al., 2008			17.4 ± 2.1	32	1–3.	12	4	5	5–10	75–85%							CMJ (W)	928.3	229.1	1145.4	285.9	23.4
Riviere et al., 2017	T	M	17.8 ± 0.9	Traditional: 8	2	6	3–6	6	2–4	70–90%	Bench press	105.6	23.3	110.6	24.7	4.7						
				Variable: 8							Bench press	95.6	9.6	100.6	10.9	5.2						
Rodriguez-Rosell et al., 2016			12.6 ± 0.5	15	2	6	2–3	4	4–8	45–60%	Squat (U 13)	38.6	17.9	57.2	15.9	48.2	10 m Sprint (U13)	1.9	0.6	1.8	0.7	3.2
			14.6 ± 0.5	14							Squat (U 15)	64.0	14.5	81.7	16.6	27.7	20 m Sprint (U13)	3.4	0.1	3.3	0.1	2.7
																	CMJ (U13)	26.6	4.3	29.8	3.9	12.0
																	10 m Sprint (U15)	1.8	0.6	1.8	0.6	1.7
																	20 m Sprint (U15)	3.1	0.1	3.1	0.1	1.3
																	CMJ (U15)	32.4	5.2	35.7	6.1	10.2
Sadres et al., 2001		MF	9.2 ± 0.3	27	2	84	1–4	3–6	5–30	30–70%	KE	18.0	5.0	31.3	7.0	73.9						
											KF	9.0	4.0	16.8	4.5	86.7						
Sander et al., 2013	T	M	13.1	18	2	104	5	5	4–10	4–10 RM	Back squat	25.0	9.6	90.0	13.5	260.0						
											Front squat	21.4	8.5	81.4	14.4	280.4						
Santos and Janeira, 2012	T	M	14.5 ± 0.6	15	3	10	2–3	6	10–12	10 RM							Med ball throw 3 kg	3.4	0.4	3.7	0.4	7.6
																	Squat jump	24.8	3.3	27.9	4.0	12.5
																	CMJ	33.3	4.3	36.7	4.2	10.2
																	Depth jump	34.8	4.1	38.1	4.3	9.5
Sarabia et al., 2015	T	M	15.0 ± 1.0	11	2	11	3–6	2			Half Squat	627.9	183	685.1	182	9.1	CMJ	31.2	3.6	32.5	2.3	4.1
											Bench press	328.0	42	341.1	49	4.0	Squat jump	28.5	3.6	31.2	2.3	9.6
																	Med ball throw	9.4	1.0	10.6	1.0	13.1
Sewall and Micheli, 1986	NR	MF	10–11	10	3	9	3	3	10	50–100%	Isom KE	19.8		24.1		21.7						
											Isom KF					12.6						
											Isom SE	16.3		21.2		30.1						
											Isom SF	5.8		7.7		32.8						
Siegel et al., 1989	U	MF	8.4 ± 0.5	50	3	12	Var	Var	Var	Var	Boys (*N* = 26)											
											Cable flexion	11.4	2.3	11.3	2.3	–0.9						
											Cable extension	12.7	2.5	12.6	2.5	–0.8						
											Handgrip right	13.4	3.1	14.9	3.3	11.2						
											Handgrip left	12.8	3.2	14.0	3.2	9.4						
											Chin up	2.4	2.5	3.8	3.6	58.3						
											Girls (*N* = 24)											
											Cable flexion	11.2	1.7	11.8	1.9	5.4						
											Cable extension	10.1	2.3	9.3	2.0	–7.9						
											Handgrip right	10.5	2.0	11.9	2.7	13.3						
											Handgrip left	9.9	2.1	11.3	2.6	14.1						
											Chin up	1.2	1.6	1.8	1.9	50.0						
Steele et al., 2017	U	MF	14.0 ± 1.0	17	2	9	2	8	4–6	4–6 RM	Bench press	31.4	7.0	36.0	2.8	14.6						
	U	MF	14.0 ± 1.0	16	2	9	2	8	12–15	12–15 RM	Bench press	30.9	7.0	35.3	2.8	14.2						
Teng et al., 2008			14.0 ± 1.0	12	2	12	3		10		Isok KF	54.0	18.0	57.0	16.0	5.6						
											Isok KE	106.0	20.0	118.0	26.0	11.3						
Tran et al., 2015	T	MF	14.0 ± 1.1	10	2	7	3	6	5–12		Isom mid thigh pull					12.7	CMJ					5.7
Tsolakis et al., 2004	U	M	11.8 ± 0.8	9	3	8	3	6	10	10 RM	Isom EF	85.1	8.3	100.2	8.4	17.7						
											Isot EF	3.2	1.6	4.0	1.5	24.2						
Velez et al., 2010	PE	MF	16.1 ± 0.2	13	3	12	2–3	12	10–15		10 RM bench press	42.0	19.2	49.5	19.8	17.9						
											10 RM seated row	61.5	21.9	71.0	24.7	15.4						
											10 RM shoulder press	38.0	21.3	49.3	4.7	29.7						
											10 RM squat	105.0	33.5	152.1	52.8	44.9						
Weakley et al., 2017	T	M	16.9 ± 0.4	35	1	12		5			Squat	77.4	32.6	96.0	18.6	24.0	10 m Sprint	1.9	0.1	1.9	0.1	–0.5
											Bench press	68.5	12.8	75.2	10.6	9.8	40 m Sprint	5.8	0.2	5.8	0.2	0.7
																	CMJ	33.8	5.2	36.2	5.6	0.7
Weltman et al., 1986	T	M	8.2 ± 1.3	16	3	14		10	30 sec		KF 30°·s	19.5	5.4	24.1	7.5	23.6	Long jump	124.8	14.3	128.6	19.2	3.0
											KF 90°·s	16.2	3.8	19.6	6.3	21.0	CMJ	21.1	4.8	23.3	3.4	10.4
											KE 30°·s	26.9	10.3	33.5	12.2	24.5						
											KE 90°·s	23.6	9.1	28.0	13.1	18.6						
											EF 30°·s	11.3	3.7	14.6	5.5	29.2						
											EF 90°·s	10.1	4.0	13.8	5.7	36.6						
											EE 30°·s	11.5	3.3	15.2	3.6	32.1						
											EE 90°·s	11.2	3.2	13.3	3.3	18.5						
Wong et al., 2010		M	13.5 ± 0.7	28	2	12	3	7–10	5–15								CMJ	55.5	6.6	58.8	7.3	5.9
																	10 m Sprint	2.1	0.2	2.0	0.1	4.8
																	30 m Sprint	4.9	0.3	4.7	0.3	2.2

*%Δ, Percent change from pre-test to post-test; BPT, balance training before plyometric training; BW, bodyweight; cm, centimeter; CMJ, counter movement jump; CSTS, core strength training on stable surface; CSTU, core strength training on unstable surface; EE, elbow extension; EF, elbow flexor; ET, elastic tubing; Ex, exercises; FFM, fat free mass; Freq, frequency; FW, free weight; Int, intensity; Isok, isokinetic; Isom, isometric; Isot, isotonic; KE, knee extension; KF, knee flexion; kg, kilogram; m, meter; Med, Medicine; Mod, moderate; MVIC, maximal voluntary isometric contraction; N, number of participants; PBT, plyometric training before balance training; PE, physical education students; PHV, peak height velocity; Post, post-test; Power, power measures; Pre, pre-test; PT, peak torque; Reps, repetitions; RM, repetition maximum; RPE, rating of perceived exertion; s, second; SD, standard deviation; Strength, strength measures; T, trained youth; TMS, trunk muscle strength; Tr, training status; U, untrained youth; Var, varied; Wks, weeks*.

*Additional Citations for Table 2A are found in the text reference list (Hettinger, 1958; Funato et al., 1986; Sewall and Micheli, 1986; Weltman et al., 1986; Blimkie, 1989; Ozmun et al., 1994; DeRenne et al., 1996; Gorostiaga et al., 1999; Sadres et al., 2001; Flanagan et al., 2002; Pikosky et al., 2002; Tsolakis et al., 2004; Drinkwater et al., 2005; Benson et al., 2007; Faigenbaum et al., 2007a, 2014, 2015; Channell and Barfield, 2008; Rhea et al., 2008; Teng et al., 2008; Chelly et al., 2009; Dorgo et al., 2009; Lubans et al., 2010; Velez et al., 2010; Wong et al., 2010; Ebada, 2011; Granacher et al., 2011a,b, 2014, 2015; Ignjatovic et al., 2011; Muehlbauer et al., 2012; Santos and Janeira, 2012; Moore et al., 2013; Moraes et al., 2013; Sander et al., 2013; Coskun and Sahin, 2014; Ferrete et al., 2014; Pesta et al., 2014; Piazza et al., 2014; Dalamitros et al., 2015; Gonzalez-Badillo et al., 2015; dos Santos Cunha et al., 2015; Sarabia et al., 2015; Tran et al., 2015; Eather et al., 2016; Harries et al., 2016; Lloyd et al., 2016; Negra et al., 2016; Prieske et al., 2016; Rodriguez-Rosell et al., 2016; Contreras et al., 2017; Steele et al., 2017; Weakley et al., 2017)*.

**Table 2B:** Power (plyometric) resistance training program descriptions.

**Article**	**Tr**	**Sex**	**Age**	***N***	**Freq**	**Wks**	**Sets**	**Ex**	**Reps**	**Int**	**Strength**	**Pre**	***SD***	**Post**	***SD***	**% Δ**	**Power**	**Pre**	***SD***	**Post**	***SD***	**% Δ**
Alves et al., 2016	U	MF	10.9 ± 0.5	45	2	8	2–3	6	4–8								1 kg Ball throw	3.6	0.6	3.8	0.6	5.6
																	3 kg Ball throw	2.2	0.4	2.4	0.4	9.1
																	Single leg jump	1.3	0.2	1.4	0.2	7.7
																	CMJ	0.2	0.0	0.2	0.0	0.0
Arabatzi, 2016	U	MF	9.3 ± 0.6	12	3	4	10	3	8–12								CMJ	18.8	0.5	21.0	0.5	11.7
																	Drop Jump	20.7	0.4	22.7	0.5	9.9
Attene et al., 2015	T	F	14.9 ± 0.9	18	2	6	2	5	6								CMJ	26.9	3.6	30.0	3.7	11.3
																	Squat jump	22.7	3.2	26.2	3.6	15.4
Borges et al., 2016	T	M															5 m Sprint	1.0	0.6	1.1	0.7	3.9
																	30 m Sprint	4.2	0.9	4.3	0.2	0.7
Buchheit et al., 2010	T	M	14.5 ± 0.5	8	1	10	4–6	4–6									10 m	1.9	0.1	1.9	0.1	0.5
																	30 m	4.7	0.3	4.6	0.2	1.9
																	CMJ	35.4	7.8	40.6	8.8	14.7
Chaabene and Negra, 2017	T	M	LPT: 12.7 ± 0.2	13	2	8	5–6		10–15								LPT: 5 m sprint	1.19	0.04	1.1	0.06	–7.5
			HPT: 12.7 ± 0.3	12	2	8	9–13		12–15								HPT: 5 m sprint	1.2	0.1	1.16	0.09	–3.3
																	LPT: 30 m sprint	4.98	0.12	4.84	0.17	–2.8
																	HPT: 30 m sprint	5.17	0.34	5.03	0.34	–2.7
Chelly et al., 2015	T	M	11.9 ± 1.0	14	4	10	3–10	6	3–10								Squat jump	0.2	2.8	0.2	0.0	14.3
																	CMJ	0.2	0.0	0.3	0.0	8.7
																	Drop jump	0.2	0.0	0.3	0.0	13.6
Cossor et al., 1999	T	M	11.7 ± 1.2	19	3	20	2	15	10–15													
																	Vertical jump	199.7	65.8	212.5	59.1	6.4
de Hoyo et al., 2016	T	M	SQ: 18 ± 1.0	9	2	8	1–3	8	2–3								CMJ	35.5	4.3	37.9	3.6	6.8
			RS: 17 ± 1.0														20 m Sprint	3.0	0.1	3.0	0.1	0.3
			PL: 18 ± 1.0														50 m Sprint	6.6	0.2	6.5	0.3	1.4
Diallo et al., 2001		M	12.3 ± 0.4	10	3	10		3														
																	CMJ	29.2	3.9	32.6	3.4	11.6
																	Squat jump	27.3	4.0	29.3	3.3	7.3
																	Running velocities 20 m (m/sec)	5.6	0.1	5.7	0.2	2.7
Faigenbaum et al., 2007b	T	M	13.4 ± 0.9	13	2	6	1–2	10–12	6–10								VJ	43.1	8.4	46.5	9.2	7.9
																	Long jump	181.1	25.9	191.9	28.5	6.0
																	9.1 m sprint	2.2	0.1	2.2	0.2	0.0
																	Ball toss	319.2	96.9	358.4	85.2	12.3
Faigenbaum et al., 2009	PE	MF	9.0 ± 0.9	40	2	9	1	12–14	6		Curl up	29.1	10.7	31	9.9	6.5	Long jump	132.0	27.5	139.9	27.0	6.0
							1		8		Push up	4.6	5.6	8.7	9.5	89.1						
							1		10													
Fernandez-Fernandez et al., 2016	T	M	12.5 ± 0.3	30	5	8	2–4	6–8	10–15								CMJ	30.1	4.3	32.0	4.1	6.3
																	5 m Sprint	1.2	0.1	1.1	0.1	5.1
																	20 m Sprint	3.5	0.2	3.4	0.2	3.7
																	Long jump	184.0	11.7	200.0	17.3	8.7
																	Medicine ball throw	626.0	91.6	680.0	114	8.6
Granacher et al., 2015	T	M	15.0 ± 1.0	12	2	8	3–5	16	5–8								CMJ IPT	44.1	4.4	46.1	3.8	4.5
																	CMJ SPT	41.1	4.2	46.4	4.9	12.9
																	Drop jump IPT	28.9	3.9	31.2	3.2	7.9
																	Drop jump SPT	27.2	4.2	30.2	2.5	11.1
																	10 m Sprint IPT	1.9	0.1	1.9	0.1	157.0
																	10 m Sprint SPT	1.9	0.1	1.9	0.1	2.1
																	30 m Sprint IPT	4.4	0.2	4.4	0.2	–0.7
																	30 m Sprint SPT	4.5	0.2	4.5	0.3	1.1
Hall et al., 2016	T	F	12.5 ± 1.7	10	2	6	1~4	20	1–6								CMJ	43.5	6.1	45.3	5.8	4.1
Hammami et al., 2016b	T	M	BPT: 12.7 ± 0.3	12	2	8	1~3	10	8–15								CMJ BPT	25.5	4.0	29.2	2.9	14.5
			PBT: 12.5 ± 0.3														CMJ PBT	24.7	2.4	26.8	1.8	8.5
																	Long jump BPT	186.0	15.9	220.7	10.3	18.7
																	Long jump PBT	177.1	11.6	206.8	13.9	16.8
																	10-m Sprint BPT	2.1	0.1	2.0	0.1	4.7
																	10 m Sprint PBT	2.1	0.1	1.9	0.2	9.5
																	30-m Sprint BPT	5.1	0.2	5.0	0.3	2.0
																	30 m Sprint PBT	5.1	0.2	5.0	0.2	2.0
Hammami et al., 2016a	T	M	15.7 ± 0.2	15	2	8	4–10	4	7–10		Dom leg PT (N–m)	41	7	46	7	12.2	5 m Sprint	1.1	0.1	1.0	0.1	7.3
Hewett et al., 1996	T	F	15.0 ± 0.6	11	3	6		16			NonDom leg PT (N–m)	37	7	46	8	24.3						
Kotzamanidis et al., 2005	T	M	17.0 ± 1.1	12	2	9	4		3–8	8,6,3 RM	Half squat	140.4	15.5	154.5	15.7	10.0	Squat jump	25.7	3.1	26.2	3.5	1.9
											Step up	65.5	7.6	76.4	7.1	16.7	DJ40	18.4	5.5	18.9	5.5	2.6
											Leg Curl	53.6	6.7	62.3	5.6	16.1	CMJ	27.2	3.4	27.5	3.3	0.9
																	30-m running speed	4.3	0.2	4.3	0.2	0.5
Kotzamanidis, 2006	U	M	11.1 ± 0.5	15	2	10	3										10 m speed (s)	2.3	0.2	2.2	0.1	2.2
																	30 m speed (s)	5.7	0.1	5.6	0.0	3.3
																	Vertical jump	23.0	4.5	31.0	4.1	34.7
King and Cipriani, 2010	T	M	FP: 15.1 ± 0.9	10	2	6	3	6	3–10								Vertical jump FP	68.1		67.3		–1.1
			SP: 15.2 ± 1.1	10													Vertical Jump SP	67.2		63.6		–5.3
Lephart et al., 2005	T	F	14.5 ± 1.3	14	3	8		11	10		Quads PT 60°/s (%BW)	211.8	45.2	227.6	23.9	7.5						
											Hams PT 60°/s (%BW)	106.3	32.6	112.7	14.4	6.0						
											Quads PT 180°/s (%BW)	128.5	22.9	147.2	18.1	14.6						
											Hams PT 180°/s (%BW)	88.4	23.7	83.6	16.3	–5.4						
											Hip abd isom PT (%BW)	169.4	34.1	165.5	35.6	–2.3						
Lloyd et al., 2012	GE9	M	9.4 ± 0.5	20	2	4	2–4	5	3–10								Hopping reactive index					
	GE 12		12.3 ± 0.3	22													GE9	0.90	0.25	0.90	0.24	0.0
	GE 15		15.3 ± 0.3	20													GE12	0.91	0.24	1.01	0.26	11.0
																	GE15	1.46	0.28	1.52	0.26	4.1
Lloyd et al., 2016	PE	M	12.7 ± 0.3	10	2	6	2	4	3–10								10 m sprint pre-PHV	2.3	0.2	2.2	0.2	4.3
			16.4 ± 0.2	10													20 m sprint pre-PHV	3.4	0.2	3.3	0.2	2.9
																	Squat jump pre-PHV	24.6	4.9	28.3	4.6	15.0
																	10 m sprint post-PHV	1.9	0.1	1.0	0.1	47.4
																	20 m sprint post-PHV	2.7	0.3	2.6	0.3	3.7
																	Squat jump post-PHV	32.3	6.4	32.7	6.3	1.2
Marques et al., 2013	T	M	13.4 ± 1.4	26	2	6	2–6	8	8–30								CMJ					7.7
																	30 m Sprint					1.7
Martel et al., 2005	T	F	15.0 ± 1.0	10	2	6	1	7	2–5		Isok PT Quad 60°	108	29	120	25		CMJ	33.4	4.7	37.1	4.5	11.0
											PT Hamstrings 60°	69	13	79	12							
											PT Quad 180°	61	17	69	21							
											PT Hamstrings 180°	48	13	56	10							
Marta et al., 2014		M	10.8 ± 0.4	76	2	8	2–6	8	3–30								1 kg Ball throw T1	333.5		355.7		6.7
																	3 kg Ball throw T1	213.3		233.2		9.3
																	Standing long Jump T1	126.8		133.8		5.6
																	CMJ T1	21.4		22.6		5.5
																	20 m Sprint T1	4.3		4.2		2.5
																	1 kg Ball throw T2	370.2		387.5		4.7
																	3 kg Ball throw T2	240.7		256.3		6.5
																	Standing long Jump T2	121.4		127.0		4.6
																	CMJ T2	20.9		22.0		5.1
																	20 m Sprint T2	4.4		4.4		1.8
Matavulj et al., 2001	T	M	15–16		3	6	3	1	10													
	DJ 50 cm			11							DJ 50 cm					0.3	DJ 50 cm					4.8
	DJ 100 cm			11							DJ 100 cm					0.03	DJ 100 cm					5.6
McCormick et al., 2016	T	F																				
	FP		16.3 ± 0.7	7	2	6	4	9	6								CMJ FP	48.3	5.4	50.1	5.3	3.8
	SP		15.7 ± 0.7	7													CMJ SP	47.7	7.1	52.6	9.4	10.3
																	Standing long Jump FP	176.9	18.5	187.1	14.2	6.0
																	Standing long Jump SP	177.9	30.1	191.9	29.1	7.9
Meylan and Malatesta, 2009	T	M	13.3 ± 0.6	14	2	8	2–4	4	6–12	1–5							SJ	30.1	4.1	30.5	3.2	0.6
																	CMJ	34.6	4.4	37.2	4.5	7.9
																	10 m Sprint	1.96	0.07	1.92	0.1	2.1
Michailidis et al., 2013	T	M	10.7 ± 0.7	24	2	12	2–4	4	5–10		10 RM Squat						30 m Sprint					–3.0
																	CMJ					27.6
																	SJ					23.3
																	DJ					15.9
Moran et al., 2016	T	M	12.6 ± 0.7	9	2		1	3	5–10								CMJ Pre-PHV	28.0	4.0	28.1	4.0	0.4
			14.3 ± 0.6	8													CMJ Mid-PHV	32.5	6.0	32.8	3.7	0.9
																	10 m Sprint Pre-PHV	2.3	0.1	2.3	0.1	0.4
																	10 m Spring mid-PHV	2.2	0.2	2.1	0.1	2.3
																	30 m Sprint pre-PHV	5.5	0.3	5.4	0.3	0.5
																	30 m Sprint mid-PHV	5.0	0.3	4.9	0.3	0.4
Noyes et al., 2012	T	F	14–17	57	3	6	1	17	5								VJ	26.2	12.3	28.5	12.0	8.8
																	18 m Sprint	3.5	0.3	3.5	0.4	0.3
Noyes et al., 2013	T	F	15.0 ± 1.0	62	3	6	1	17	5								37 m Sprint	6.1	0.4	6.0	0.4	2.0
																	VJ 2 Step	40.7	8.9	42.1	8.3	3.4
																	CMJ	32.9	6.7	32.6	25.8	–0.9
Noyes et al., 2012	T	F	14.5 ± 1.0	34	3	6	1	17	5–25		Sit-up (reps)	37.7	5.3	40.5	5.9	7.4	CMJ	40.1	7.1	41.5	4.5	3.5
Pereira et al., 2015	T	M	14.0	10	2	8	2	5	8–20								CMJ	26.9	4.5	32.3	9.0	20.1
																	Medicine ball throw	7.5	15.2	7.9	14.3	5.2
Piazza et al., 2014	T	F	11.9 ± 1.0	18	2	6	1	10	3													
																	SJ	410.4	41.6	421.5	28.4	2.7
																	CMJ	457.2	30.6	485.0	33.8	6.1
Potdevin et al., 2011	T	M	14.3 ± 0.2	12	2	6	2–10	13	4–12								CMJ	28.9	4.8	32.5	4.2	12.2
																	SJ	26.2	3.8	31.1	4.9	18.9
Ramirez-Campillo et al., 2013	T	M	13.2 ± 1.8	38	2	7	2	3	10								CMJ	27.0	5.8			4.3
																	20 m Sprint	4.3	0.6			0.4
Ramirez-Campillo et al., 2014	T	M	13.2 ± 1.8	38	2	7	2	7	10	High							CMJ	26.7	4.7			2.2
																	20 m Sprint	4.39	0.48			3.7
Ramirez-Campillo et al., 2015a	T	M	11.6 ± 2.7	12	2	6	2	6	5–10								30 m sprint	6.0	0.6			6.5
																	CMJ	30.5	9.3			15.4
																	Horizontal jump	153.0	4.1			14.6
Ramirez-Campillo et al., 2015b		M	NPPT: 13.0 ± 2.1	8	2	6	2	2	5								Vert CMJ w/arms					
			PPT: 12.8 ± 2.8	8			2		5–10								NPPT	28.5	10.4			10.9
																	PPT	27.9	8.7			16.6
																	Horz CMJ w/arms					
																	NPPT	163.0	42.6			4.6
																	PPT	160.0	27.9			7.9
																	Right leg horiz CMJ w/arms					
																	NPPT	138.0	35.3			2.8
																	PPT	138.0	27.7			13.5
																	Left leg horiz CMJ w/arms					
																	NPPT	136.0	42.9			14.1
																	PPT	134.0	27.0			21.2
																	Maximal kicking velocity					
																	NPPT	68.3	15.4			5.7
																	PPT	67.1	16.3			10.1
																	10 m sprint time					
																	NPPT	2.6	0.4			0.9
																	PPT	2.7	0.3			1.6
Ramírez-Campillo et al., 2015c	T	M	BG: 11.0 ± 2.0	12	2	6	2–3	6	5–10								CMJ:					
			UG: 11.6 ± 1.7	16													BG	31.1	2.0			18.7
			BUG: 11.6 ± 2.7	12													UG	29.5	4.3			7.9
																	BUG	30.5	9.3			15.4
																	Horizontal CMJ					
																	BG	166	33			17.4
																	UG	153	22			8.9
																	BUG	153	41			14.6
																	Maximal kicking velocity					
																	BG	59.2	18.4			8.4
																	UG	59.9	10.8			14.0
																	BUG	61.8	19.6			12.0
																	30 m Sprint					
																	BG	5.7	0.5			–3.2
																	UG	6.1	0.4			–6.2
																	BUG	6.0	0.6			–6.5
Rosas et al., 2016	T	M	12.3 ± 2.3	21	2	6		6	4–8								CMJ	31.7	9.0			4.3
																	Horizontal jump	159.0	35.7			6.1
Santos and Janeira, 2012	T	M	15.0 ± 0.5	14	2	10	2–4	6	6–15								Squat jump	25.2	3.5	29.2	4.1	15.8
																	CMJ	30.3	4.3	34.5	5.0	13.8
																	Medicine ball throw	3.4	0.4	3.9	0.4	14.9
Santos et al., 2012	U	M	13.3 ± 1.0	30	2	8	1–5	7–8	3–8								GR Group					
																	CMJ	0.3	0.1	0.3	0.1	4.4
																	Long jump	1.5	0.3	1.6	0.3	4.7
																	1 kg Medicine ball throw	7.5	1.7	8.2	1.6	8.7
																	3 kg Medicine ball throw	4.7	1.0	5.1	1.1	9.9
																	20 m	4.5	0.5	4.1	0.4	10.8
																	GCOM group					
																	CMJ	29.8	0.1	31.6	0.1	6.0
																	Long jump	1.7	0.3	1.7	0.3	4.2
																	1 kg Medicine ball throw	7.3	1.6	7.6	1.7	4.5
																	3 kg Medicine ball throw	4.6	1.1	5.1	1.2	11.1
																	20 m	4.4	0.6	3.8	0.3	13.0
Skurvydas et al., 2010		M	10.3 ± 0.3	13	2	8	1	1	30		MVIC	79.4	22.1	86.6	23.1	9.1	CMJ	24.1	3.8	32.8	5.1	36.1
Skurvydas and Brazaitis, 2010		M	10.2 ± 0.3	13	2	8	1	1	30								Girls	21.8	3.3	29.9	3.8	37.7
Sohnlein et al., 2014	T	M	13.0 ± 0.9	12	2	16	2–5	9	6–16	Max							10 m Sprint	1.8	0.1	1.8	0.1	2.2
																	30 m Sprint	4.4	0.2	4.3	0.1	2.5
																	5 m Sprint	1.1	0.0	1.0	0.0	3.8
																	20 m Sprint	3.2	0.1	3.1	0.1	3.2
Szymanski et al., 2007	T	M	15.4 ± 1.1	25	3	12	2	7	6–10		Dom TRS					17.1	Standing long jump	2.3	0.2	2.5	0.1	7.3
											NonDom TRS					18.3	Medicine ball hitter's throw					10.6
											Parallel squat (kg)	106.3	23.4	145	27.7	26.7						
											Bench press (kg)	71.7	15.9	86.1	15.2	16.7						
Thomas et al., 2009	T	M	17.3 ± 0.4	12	2	6				Chu's							5 m Sprint					
																	DJ trained	1.0	0.1	1.1	0.1	1.9
																	CMJ trained	1.1	0.1	1.1	0.1	0.9
																	10 m Sprint					
																	DJ trained	1.8	0.1	1.8	0.2	1.1
																	CMJ trained	1.8	0.1	1.8	0.2	0.0
																	20 m Sprint					
																	DJ trained	3.1	0.1	3.1	0.2	1.0
																	CMJ trained	3.2	0.1	3.2	0.3	0.6
Witzke and Snow, 2000	PE	F	14.6 ± 0.5	25	3	12	2–3	Str:7	8–12	5–15% BW	Leg strength	96	19.9	107.7	17.3	16.7	Leg power	392.0	82.0	445.0	91.0	13.5
						24	2–3	Plyo: 5–20	2–20	mod-high												

*%Δ, percent change from pre-test to post-test; BPT, balance training before plyometric training; BW, bodyweight; cm, centimeter; CMJ, counter movement jump; DJ, drop jump; Dom, dominant; Ex, exercises; FP, frontal plane; Freq, frequency; GCOM, combined resistance training and endurance; GR, resistance training alone; Int, intensity; IPT, plyometric training on unstable surface; Isok, isokinetic; Isom, isometric; kg, kilogram; m, meter; Mod, moderate; MVIC, maximal voluntary isometric contraction; N, number of participants; Nm, newton meter; NonDom, non-dominant; NPPT, no plyometric training; PBT, plyometric training before balance training; PE, physical education students; Pre, pre-test; PHV, peak height velocity; PL, plyometric; Post, post-test; Power, power measures; PPT, plyometric training; Reps, repetitions; RS, resisted sprinting; s, second; SD, standard deviation; SJ, squat jump; SP, sagittal plane; SPT, plyometric training on stable surface; SQ, squat; ST, Strength; Strength, strength measures; T, trained youth; Tr, training status; TRS, torso rotational strength; U, untrained youth; Wks, weeks*.

*Additional Citations for Tables 2A,B are found in the text reference list (Hewett et al., 1996; Cossor et al., 1999; Witzke and Snow, 2000; Diallo et al., 2001; Matavulj et al., 2001; Martel et al., 2005; Szymanski et al., 2007; Meylan and Malatesta, 2009; Thomas et al., 2009; Buchheit et al., 2010; King and Cipriani, 2010; Skurvydas and Brazaitis, 2010; Skurvydas et al., 2010; Potdevin et al., 2011; Santos and Janeira, 2011; Lloyd et al., 2012; Noyes et al., 2012, 2013; Santos et al., 2012; Marques et al., 2013; Michailidis et al., 2013; Ramirez-Campillo et al., 2013, 2015a,b; Marta et al., 2014; Piazza et al., 2014; Sohnlein et al., 2014; Attene et al., 2015; Chelly et al., 2015; Pereira et al., 2015; Alves et al., 2016; Arabatzi, 2016; Borges et al., 2016; de Hoyo et al., 2016; Fernandez-Fernandez et al., 2016; Hall et al., 2016; McCormick et al., 2016; Moran et al., 2016; Rosas et al., 2016)*.

### Muscle power (jump) measures

**Figure 2:**
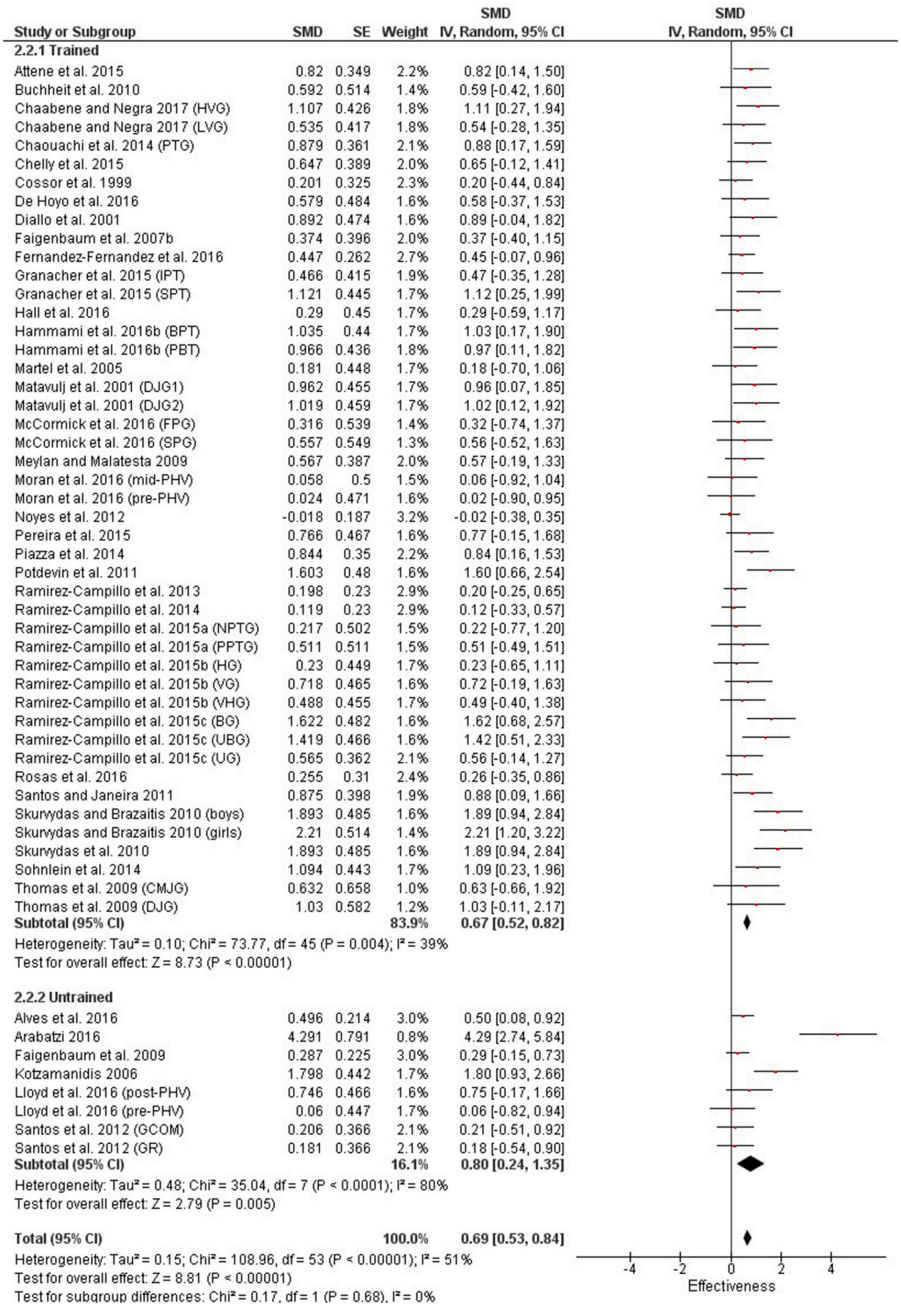
Power training effects on jump measures for trained and untrained subjects. Positive SMD values indicate performance changes from pre to post related to training effects, while negative SMDs are indicative of non-effective changes from pre to post. SMD, Standardized mean difference expresses the size of the intervention effect relative to the variability observed in that study. SE, Standard Error. Weight, proportional weight or contribution of each study to the overall analysis.

**Figure 3:**
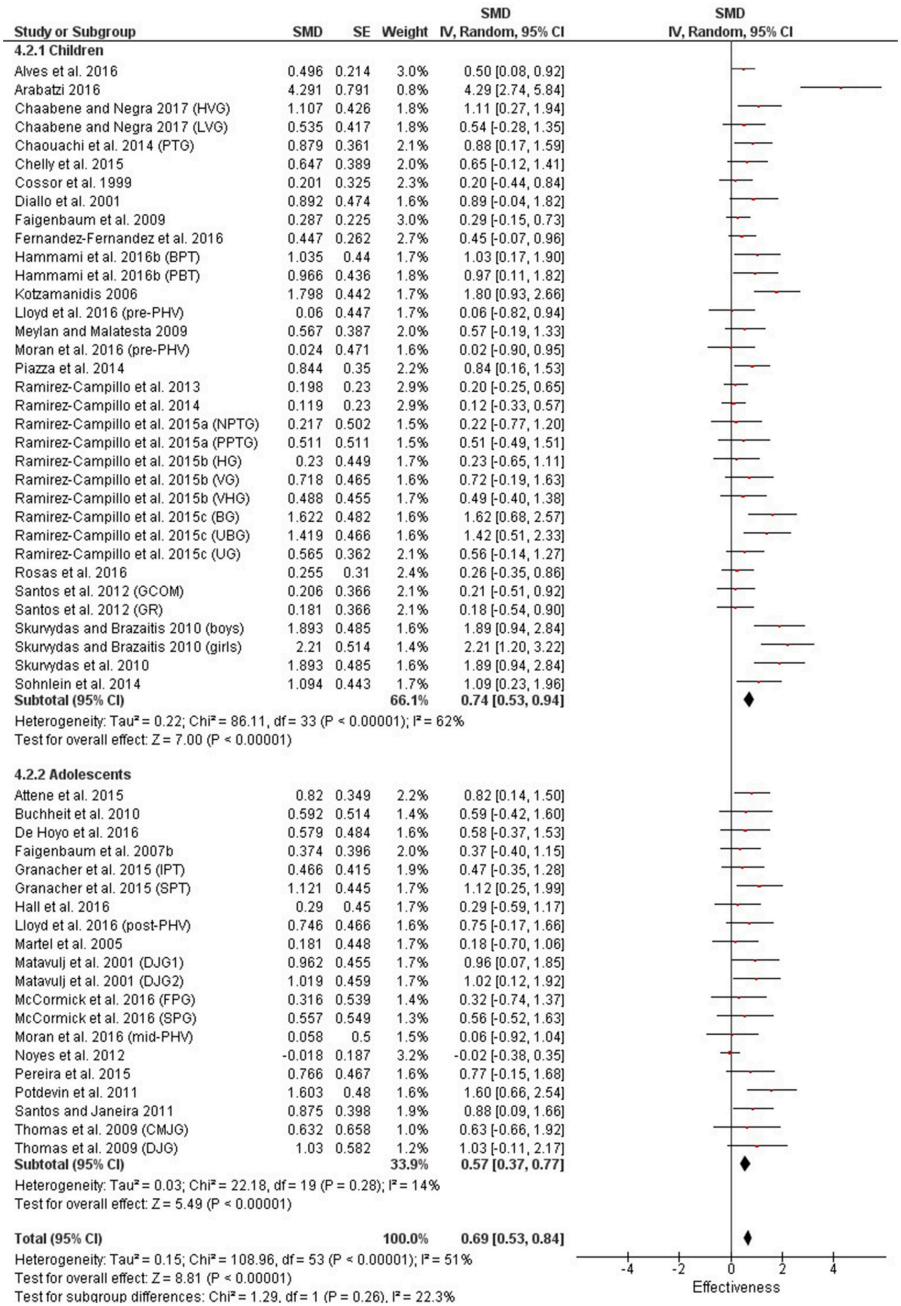
Power training effects on jump measures for children and adolescents. Positive SMD values indicate performance changes from pre to post related to training effects, while negative SMDs are indicative of non-effective changes from pre to post. SMD, Standardized mean difference expresses the size of the intervention effect relative to the variability observed in that study. SE, Standard Error. Weight, proportional weight or contribution of each study to the overall analysis.

**Figure 4:**
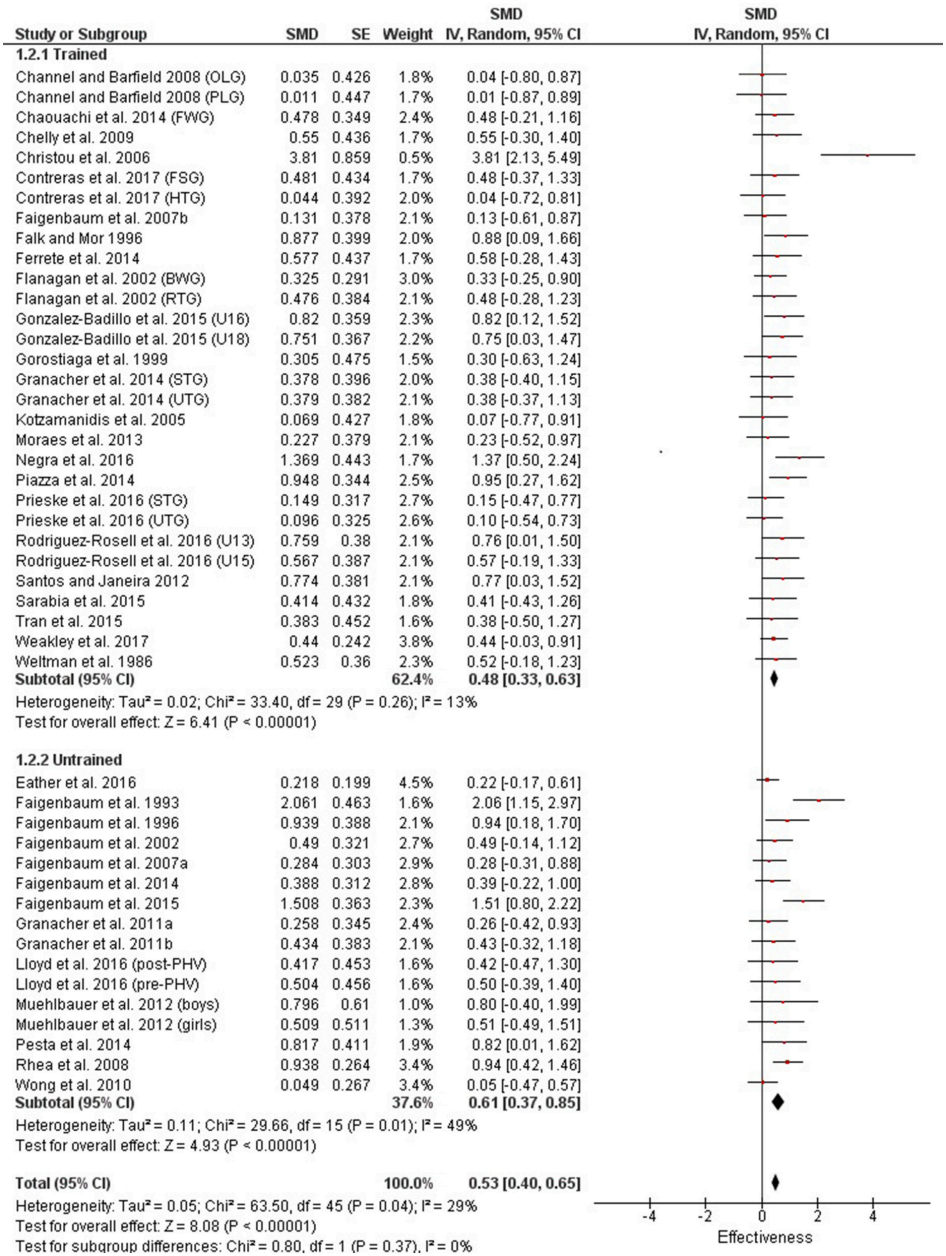
Strength training effects on jump measures for trained and untrained subjects. Positive SMD values indicate performance changes from pre to post related to training effects, while negative SMDs are indicative of non-effective changes from pre to post. SMD, Standardized mean difference expresses the size of the intervention effect relative to the variability observed in that study. SE, Standard Error. Weight, proportional weight or contribution of each study to the overall analysis.

**Figure 5:**
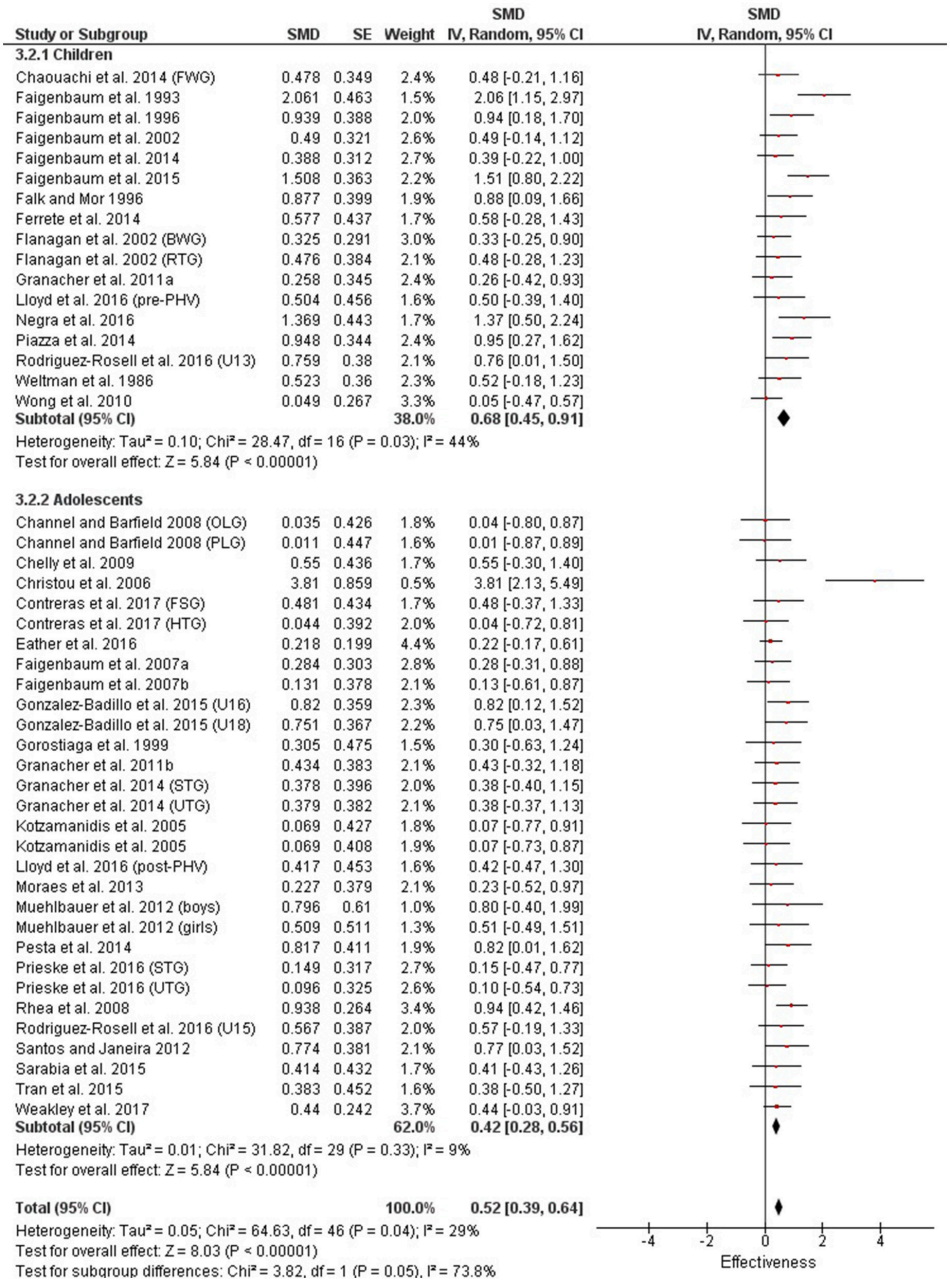
Strength training effects on jump measures for children and adolescents. Positive SMD values indicate performance changes from pre to post related to training effects, while negative SMDs are indicative of non-effective changes from pre to post. SMD, Standardized mean difference expresses the size of the intervention effect relative to the variability observed in that study. SE, Standard Error. Weight, proportional weight or contribution of each study to the overall analysis.

Table 3 shows that power (plyometric) training studies provided higher magnitude changes in jump performance than strength training studies. In terms of general descriptors, power training studies exceeded strength training studies with trained (moderate vs. small), untrained (large vs. moderate)(Figures 2, **4**) and adolescent (moderate vs. small) populations (Figures 3, **5**). For the overall or general results (Figures 2, 4) as well as with children (Figures 3, 5), the descriptive classifications were the same (moderate magnitude improvements), although the precise SMDs values were higher with power training. When comparing specific populations (power and strength training combined), untrained individuals (moderate to large magnitude) experienced greater jump height gains than trained participants (small to moderate). Similarly, with training groups combined, children experienced larger jump height gains than adolescents, although the descriptive classification only differed with strength training (moderate vs. small), but not power training.

**Table 3:** Summary of meta-analysis results.

	**General**	**Trained vs**.	**Untrained**	**Children vs**.	**Adolescents**
Power training effects on jump measures	0.69 Moderate	0.67 Moderate	**0.80 Large**	***0.74****Moderate***	*0.57 Moderate*
Strength training effects on jump measures	0.53 Moderate	0.48 Small	**0.61 Moderate**	***0.68 Moderate***	*0.42 Small*
Power training effects on sprint measures	0.38 Small	0.32 Small	1.19^*^ **Large**	***0.47****Small***	*0.13 Trivial*
Strength training effects on sprint measures	0.48 Small	0.45 Small	0.57^*^ **Moderate**	***0.73****Moderate***	*0.36 Small*
Power training effects on lower body strength measures	0.16^**^ Trivial	Not reported	Not reported	Not reported	0.16^**^ Trivial
Strength training effects on lower body strength measures	1.14 Large	1.23 Large	**1.08 Large**	***1.39 Large***	*0.88 Large*

*Shaded row values illustrate higher magnitude changes compared to the corresponding measure. Bolded values illustrate higher magnitude changes for untrained vs. trained participants. Bolded and underlined values indicate higher magnitude changes for children vs. adolescents*.

* *3 studies met inclusion criteria*;

** *4 studies met the inclusion criteria*.

### Sprint speed measures

**Figure 6:**
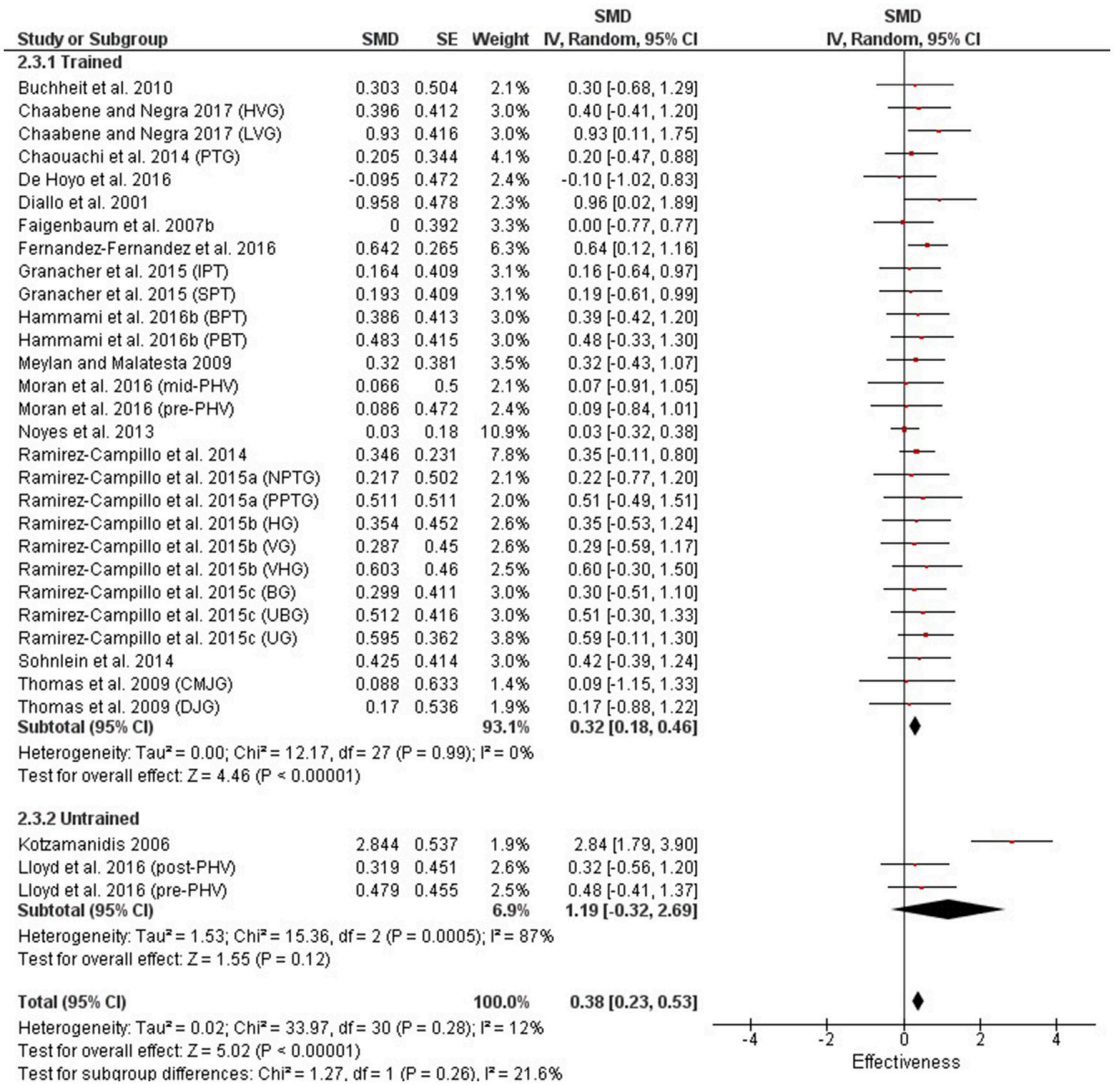
Power training effects on sprint measures for trained and untrained subjects. Positive SMD values indicate performance changes from pre to post related to training effects, while negative SMDs are indicative of non-effective changes from pre to post. SMD, Standardized mean difference expresses the size of the intervention effect relative to the variability observed in that study. SE, Standard Error. Weight, proportional weight or contribution of each study to the overall analysis.

**Figure 7:**
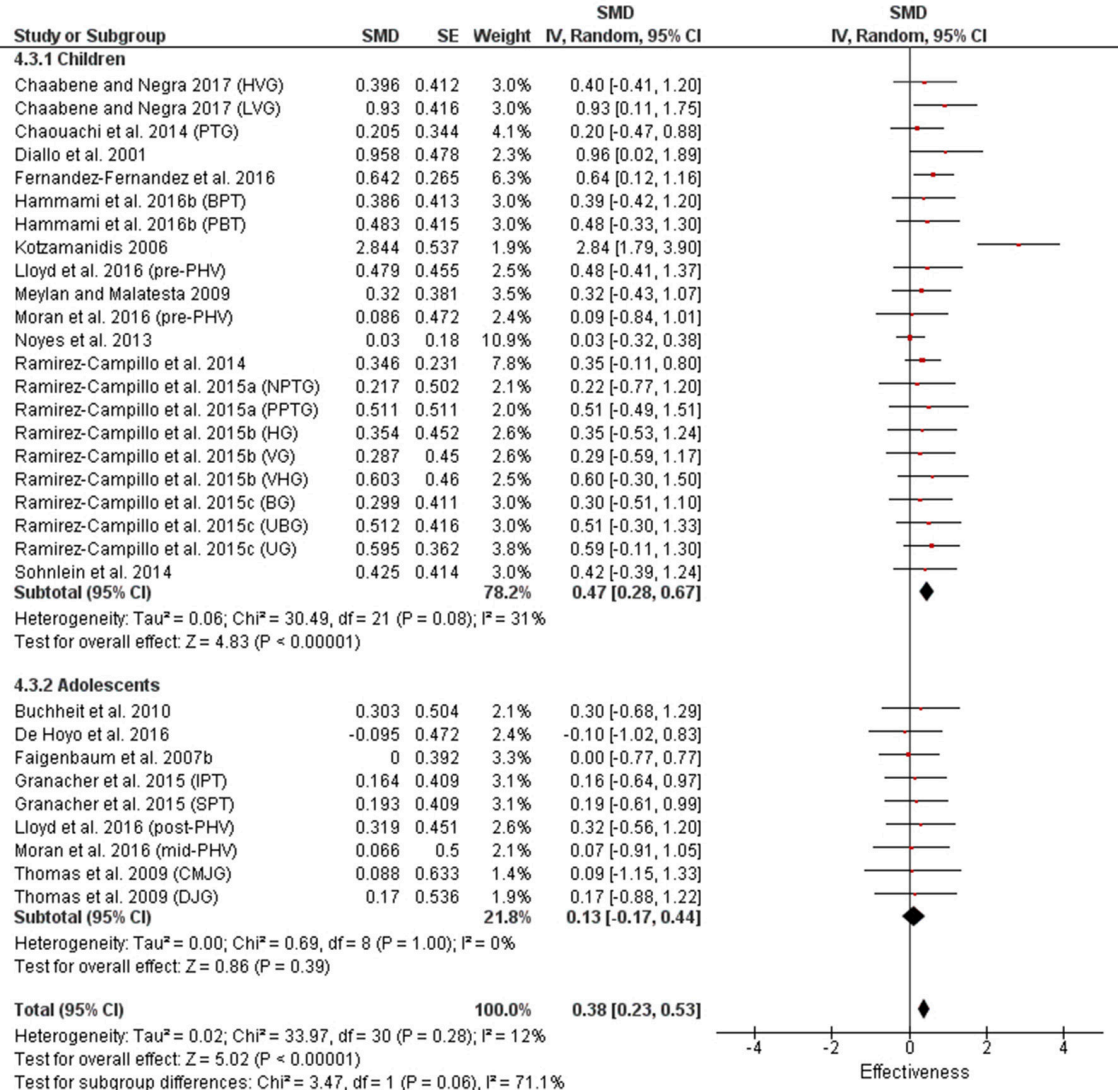
Power training effects on sprint measures for children and adolescents. Positive SMD values indicate performance changes from pre to post related to training effects, while negative SMDs are indicative of non-effective changes from pre to post. SMD, Standardized mean difference expresses the size of the intervention effect relative to the variability observed in that study. SE, Standard Error. Weight, proportional weight or contribution of each study to the overall analysis.

**Figure 8:**
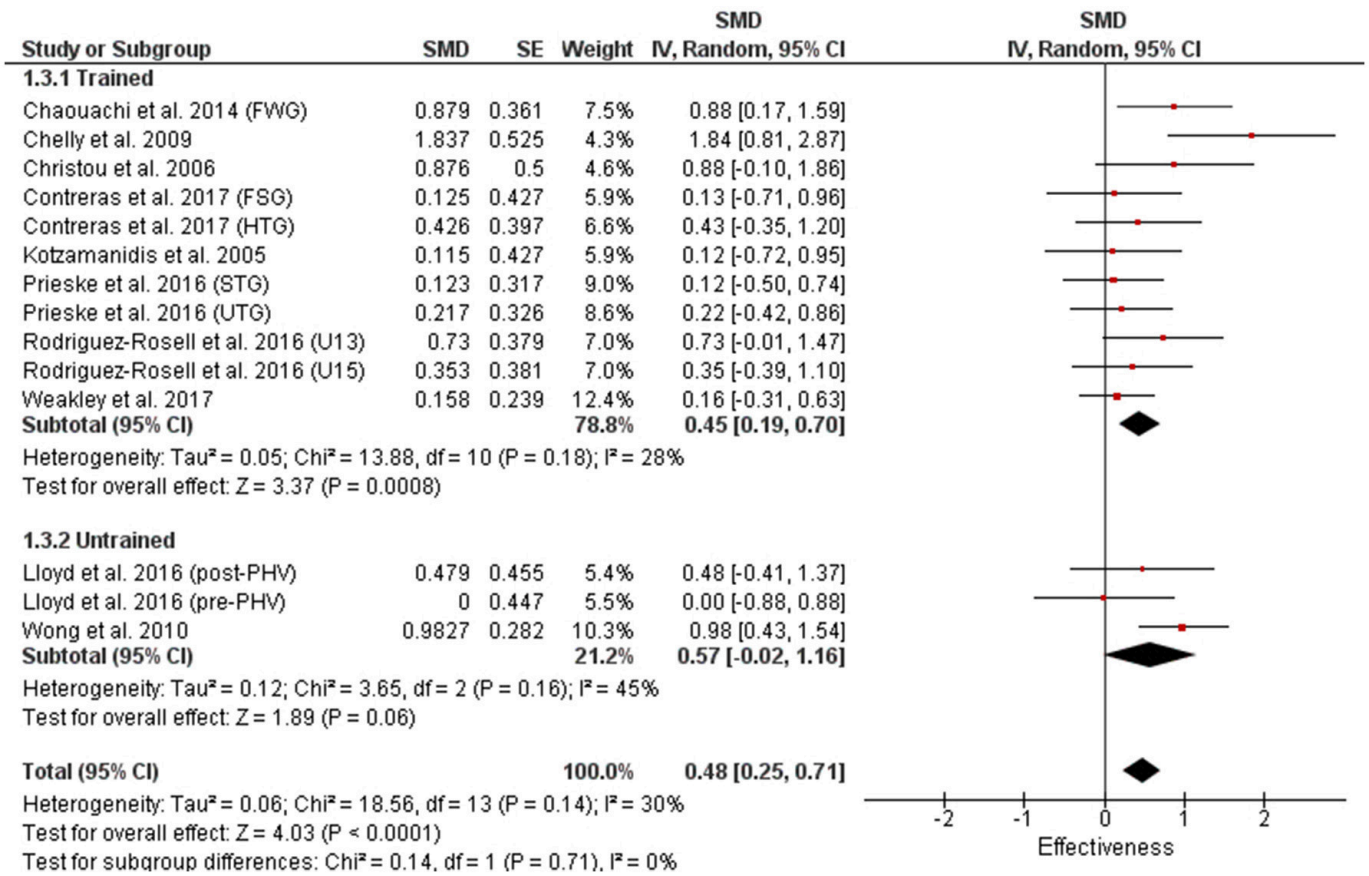
Strength training effects on sprint measures for trained and untrained subjects. Positive SMD values indicate performance changes from pre to post related to training effects, while negative SMDs are indicative of non-effective changes from pre to post. SMD, Standardized mean difference expresses the size of the intervention effect relative to the variability observed in that study. SE, Standard Error. Weight, proportional weight or contribution of each study to the overall analysis.

**Figure 9:**
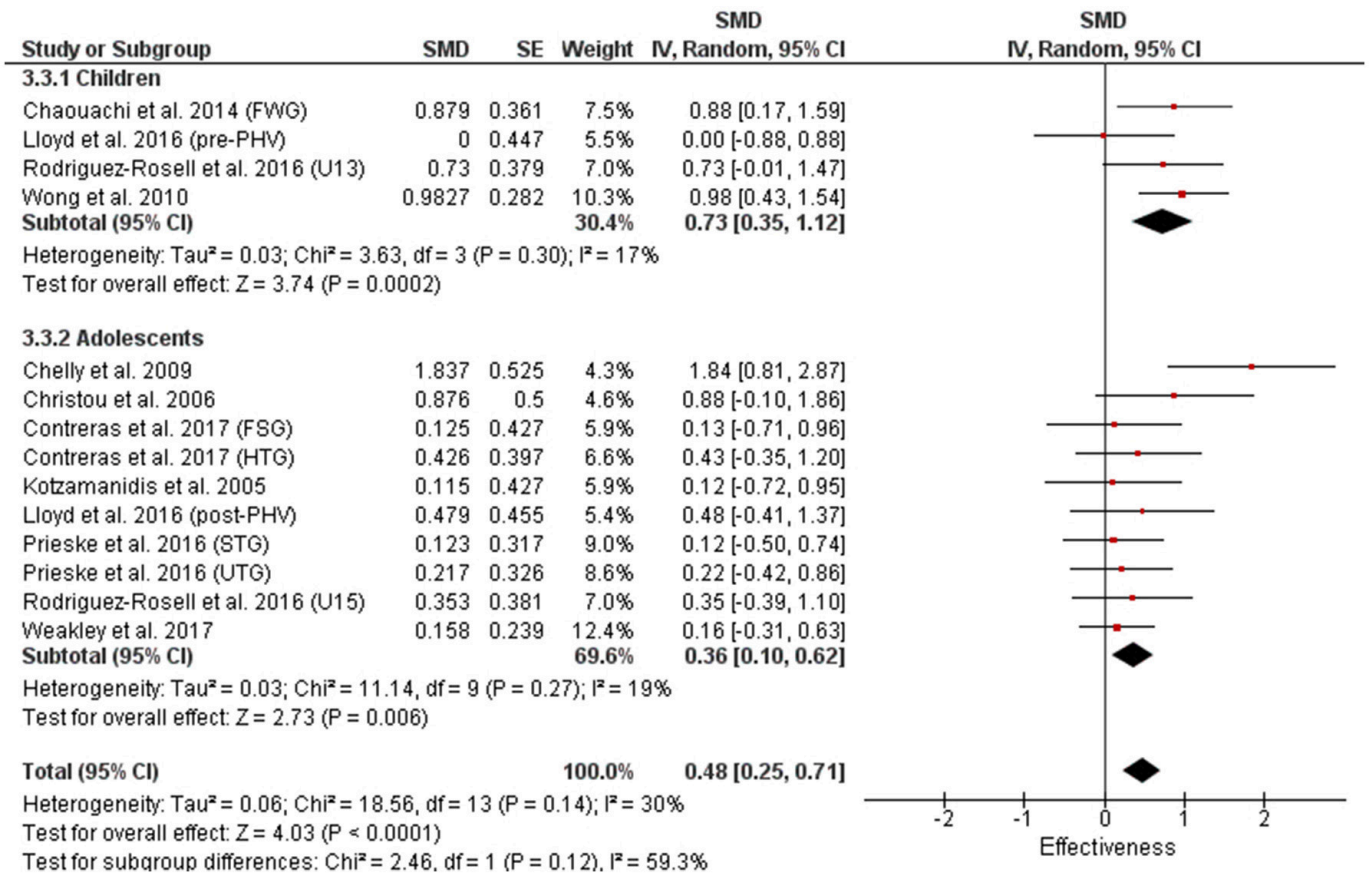
Strength training effects on sprint performance for children and adolescents. Positive SMD values indicate performance changes from pre to post related to training effects, while negative SMDs are indicative of non-effective changes from pre to post. SMD, Standardized mean difference expresses the size of the intervention effect relative to the variability observed in that study. SE, Standard Error. Weight, proportional weight or contribution of each study to the overall analysis.

In contrast to power (jump) results, strength training studies tended to provide better sprint time results than power training (Table 2). However, it was only in the children and adolescent strength vs. power training comparison where the descriptive classifications for strength training exceeded power training with moderate vs. small and small vs. trivial classifications, respectively (Figures 7, 9). In contrast, power training (only 3 measures) provided a greater magnitude change than strength training (30 measures) with untrained populations demonstrating a large vs. moderate improvement in sprint time (Figures 6, 8). Again, similar to power (jump) measures, untrained and child populations had greater magnitudes and descriptors than trained and adolescents respectively for both strength and power training.

### Muscle strength measures

**Figure 10:**
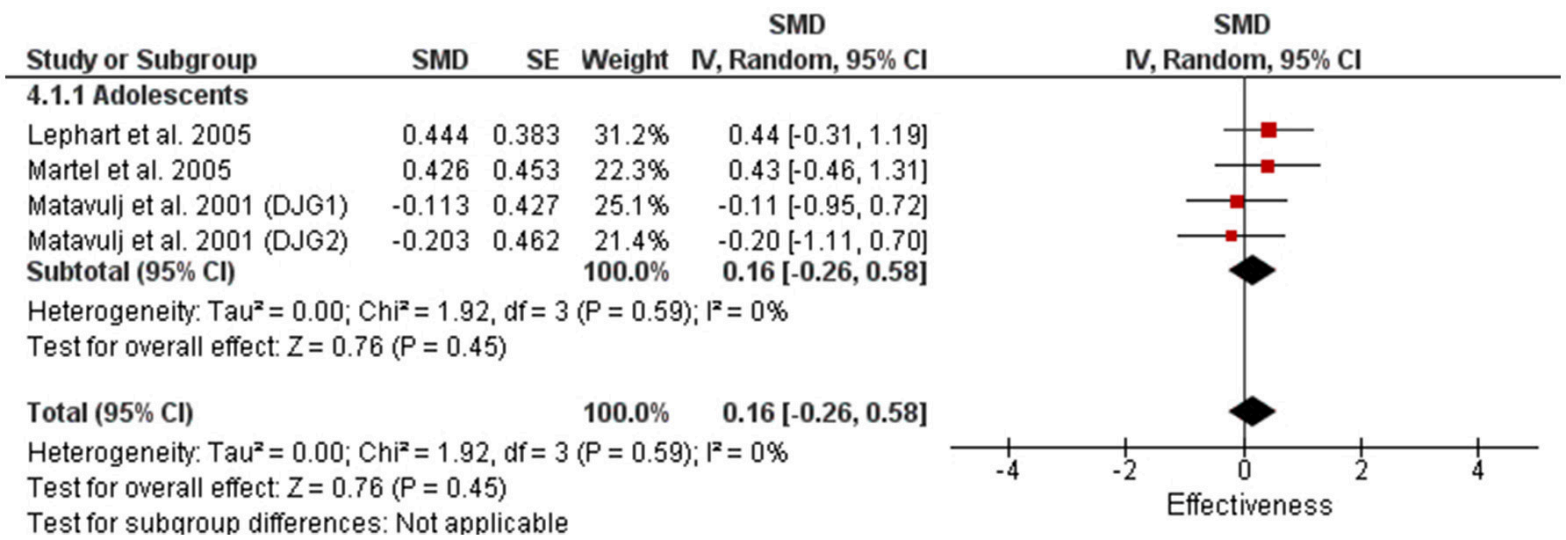
Power training effects on lower body strength for adolescents only. Positive SMD values indicate performance changes from pre to post related to training effects, while negative SMDs are indicative of non-effective changes from pre to post. SMD, Standardized mean difference expresses the size of the intervention effect relative to the variability observed in that study. SE, Standard Error. Weight, proportional weight or contribution of each study to the overall analysis.

**Figure 11:**
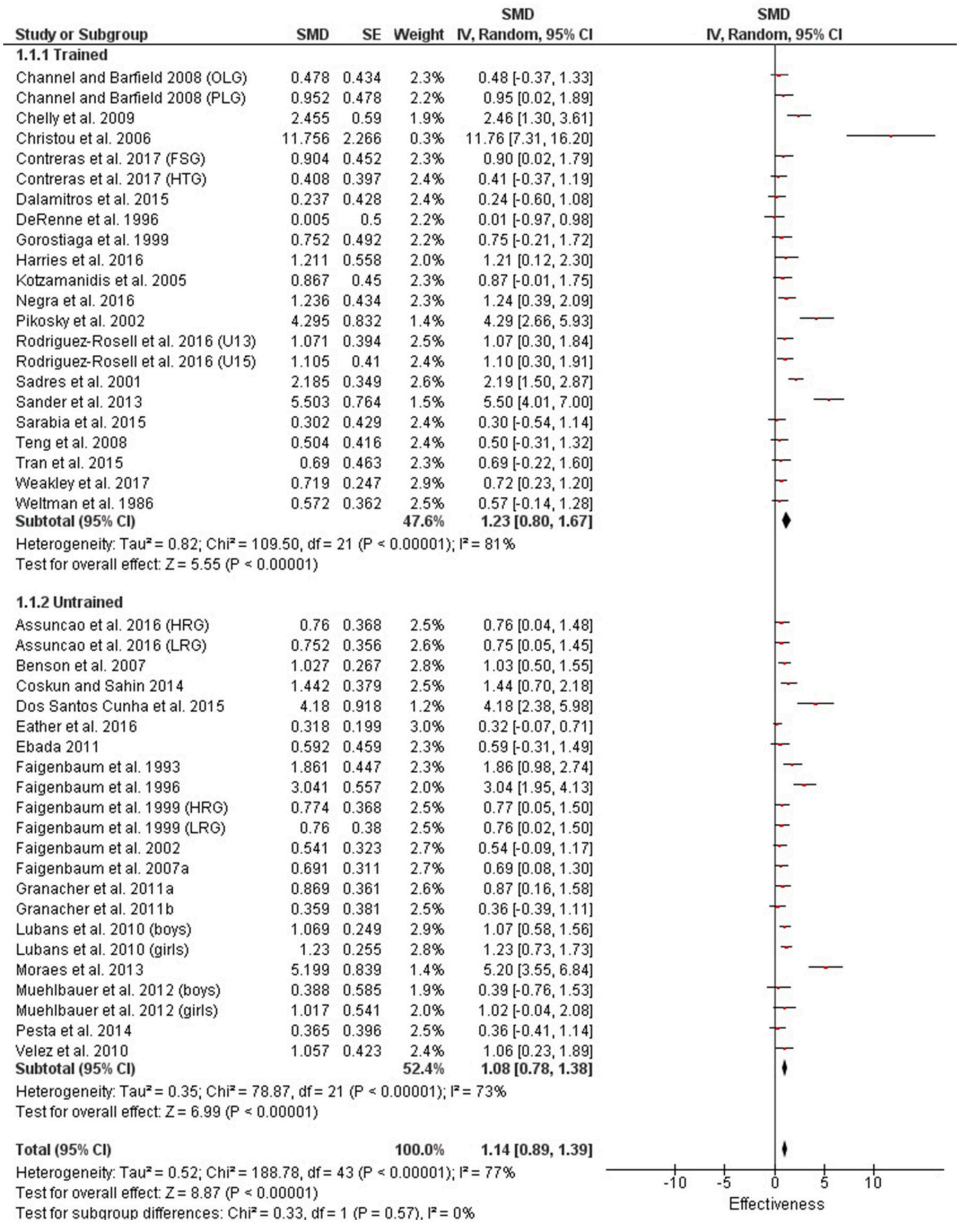
Strength training effects on lower body strength for trained and untrained subjects. Positive SMD values indicate performance changes from pre to post related to training effects, while negative SMDs are indicative of non-effective changes from pre to post. SMD, Standardized mean difference expresses the size of the intervention effect relative to the variability observed in that study. SE, Standard Error. Weight, proportional weight or contribution of each study to the overall analysis.

**Figure 12:**
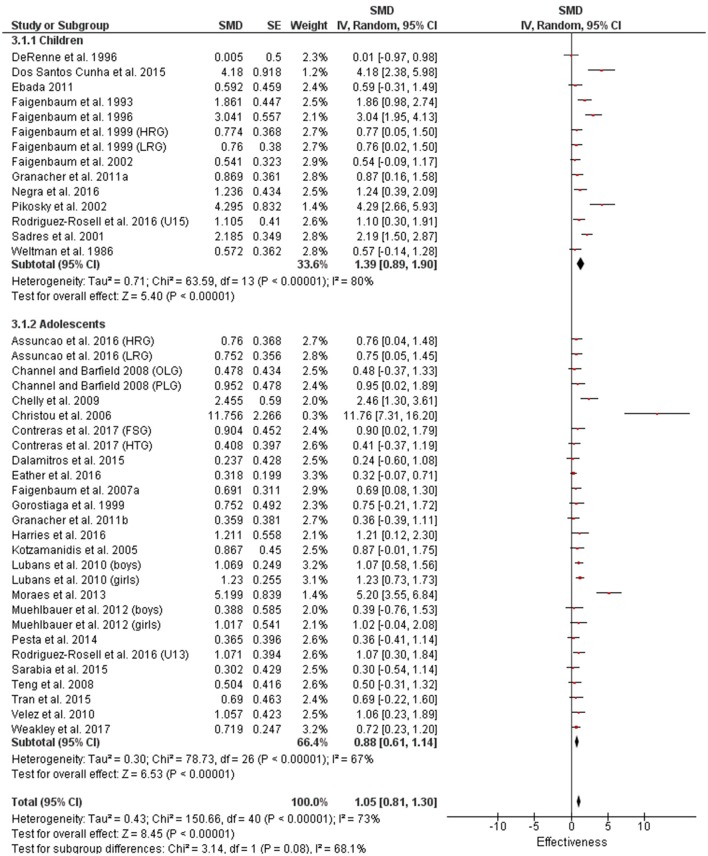
Strength training effects on lower body strength for children and adolescents. Positive SMD values indicate performance changes from pre to post related to training effects, while negative SMDs are indicative of non-effective changes from pre to post. SMD, Standardized mean difference expresses the size of the intervention effect relative to the variability observed in that study. SE, Standard Error. Weight, proportional weight or contribution of each study to the overall analysis.

There were very few power training studies that measured lower body strength with no studies that utilized children or differentiated between trained and untrained individuals (Figure 10). The 4 power training measures within our review used adolescents with only a trivial magnitude improvement compared to large magnitude improvements in all categories (0.88–1.35) with the 45 strength training measures (Figures 11, 12).

## Discussion

This is the first systematic review and meta-analysis that compared the effects of strength vs. power training on measures of muscle strength, power, and speed in trained and untrained youth. The most pertinent findings of the present study were the tendencies for training specificity with power measures (power training more effective than strength training), but a lack of training specificity with sprint measures (strength training more effective than power training) with youth. Thirdly, strength training exhibited uniformly large magnitude changes to lower body strength measures, which contrasted with the generally trivial, small and moderate magnitude training improvements of power training upon lower body strength, sprint and jump power measures, respectively. Furthermore, untrained youth displayed more substantial improvements in jump and sprint measures with both power and strength training compared to trained youth.

The greater magnitude improvements in power measures with power vs. strength training corresponds with the training specificity principle (Sale and MacDougall, 1981; Behm, 1988, 1995; Behm and Sale, 1993). Training specificity dictates that training adaptations are greater when the training mode, velocities, contraction types and other training characteristics most closely match the subsequent activity, sport or tests. The higher speed and power movements associated with power training would be expected to provide more optimal training adaptations for explosive type jump measures. Power training (e.g., plyometrics) can improve youth's ability to increase movement speed and power production (Behm et al., 2008). Chaouachi et al. (2014) reported similar findings when they compared training programs that involved two types of power training (Olympic weight lifting and plyometric) and traditional RT. In accordance with the present review and the concept of training specificity, both plyometric and Olympic weight lifting in the Chaouachi study provided greater magnitude improvements in CMJ than traditional RT.

It should be noted though, that while the numerical SMD values for power training exceeded strength training for power measures, the descriptor categorization overall was the same: moderate for both power and strength training. Thus, while it is conceded that power training demonstrates a numerical advantage over strength training for power measures (e.g., jump performance), the relative extent or degree of superiority was not overwhelming. The relative magnitude of improvement with power training (moderate to large: 0.6–0.8) for power measures (e.g., jumps) did not match the training specific extent or consistency of improvements associated with strength training on lower body strength (uniformly large: 0.88–1.35). Hence, the training specific response of strength training (strength training effects on strength measures) was consistently more substantial than the power training specific response (power training effects on jump power measures). Furthermore, power training specificity did not extend to another power and speed related measure: sprint speed.

Strength training magnitudes of change exceeded power training for sprint measures (exception of untrained participants). These findings contradict the long-held concept of training specificity (Sale and MacDougall, 1981; Behm, 1988, 1995; Behm and Sale, 1993). Slower, more deliberate movements of traditional RT would not be expected to provide optimal training adaptations for sprint measures that involve higher speed, stretch-shortening cycle (SSC) type activities. Again, similar findings were reported by Chaouachi et al. (2014) who found that traditional RT provided superior training adaptations compared to both Olympic weight lifting and plyometric training for 5 and 20 meter sprints. However, Radnor et al. (2017) reported contradictory results to the present meta-analysis with plyometric training and combined strength and plyometric training providing more positive responders than strength training alone for sprint velocity. The Radnor study incorporated school aged boys (not specifically trained) whereas the present review included both highly trained athletes and untrained youth. Similar to Radnor and colleagues, untrained youth in this meta-analysis participating in power training had greater magnitude improvements in sprint measures than trained athletes or the mean results of both populations.

One of the main factors contributing to optimal sprint performance is the capacity to generate a high rate of muscular force (Aagaard et al., 2002; Cronin and Sleivert, 2005; Cormie et al., 2007). Sprint actions employ stretch-shortening cycle (SSC) actions that involve the sequential combination of eccentric and concentric muscle contractions (Komi, 1986). SSC based actions tend to promote greater concentric force outputs when there is a rapid and efficient storage and transfer of elastic energy from the eccentric to the concentric phases (Cavagna et al., 1968; Bosco et al., 1982a,b; Cormie et al., 2010). Elastic and contractile (e.g., increased time for muscle activation, pre-load effect, muscle-tendon interaction, stretch reflexes) components affect maximal power output (Cavagna et al., 1968; Ettema et al., 1990; Lichtwark and Wilson, 2005; Avela et al., 2006). These mechanical and reflexive contributions occur over a short duration and thus the transition from eccentric to concentric phases must be brief (McCarthy et al., 2012). Reaction forces from sprints and hurdle jumps can generate reaction forces of ~4–6 times the individual's body mass (Mero et al., 1992; Cappa and Behm, 2011). Since the predominant jump measures were from bilateral CMJ and squat jumps, the ground reaction forces upon each limb would have been substantially lower (typically ½) than with high speed sprinting (with unilateral landings) (Dintiman and Ward, 2003; Cappa and Behm, 2011). The training specific related power (jump height) improvements seen with power training in this review would not necessitate similar eccentric strength capacities compared to the reaction forces experienced with sprinting. An individual who lacks sufficient eccentric strength must accommodate the eccentric forces by absorbing those forces over a longer time period, which would nullify the advantages of SSC actions (Miyaguchi and Demura, 2008). The lack of sprint training specificity with youth might be attributed to a lack of foundational eccentric (and likely concentric) strength. The effectiveness of traditional RT with youth sprinting would lie in its ability to build this essential strength component allowing youth to take advantage of the SSC mechanical and reflexive power amplification. Plyometric training would not be effective with any individual (youth or adult) who must absorb reaction forces over a prolonged period and thus cannot efficiently transfer the eccentric forces to the concentric power output.

The CMJ, drop, squat and other jumps evaluated in this meta-analysis all involved bilateral take-offs and landings. In contrast, sprinting is a series of rapid, unilateral landings and propulsions which would place greater challenges on the balance capabilities of the individual. Balance is another important contributor to SSC and sprint performance especially in youth (Hammami et al., 2016a). Balance affects force, power output and movement velocity (Anderson and Behm, 2005; Drinkwater et al., 2007; Behm et al., 2010a,b). Since balance and coordination are not fully mature in children (Payne and Isaacs, 2005), the effectiveness of plyometric training could be adversely affected. Hammami et al. (2016a) reported large-sized correlations between balance measures and proxies of power with youth (*r* = 0.511–0.827). These correlation coefficients were greatest with the more mature post-peak height velocity (PHV) youth, suggesting that the poorer postural control of the less mature pre-PHV and PHV youth had negative consequences upon power output. Similarly, significant positive correlations between maximum speed skating performance and a static wobble board balance test were reported in youth under 19 years of age (Behm et al., 2005). Thus, plyometric training activities are positively augmented with greater balance or postural control. For example, when 4 weeks of balance training was incorporated prior to 4 weeks of plyometric training the training outcomes were significantly better with youth than in the reverse order (Hammami et al., 2016b). Hence, the combination of inadequate strength and balance would inhibit positive sprint training adaptations associated with plyometric training with youth. In conflict with the training specificity principle, traditional RT may be more beneficial for promoting sprint adaptations in youth since it can build a foundation of strength upon which youth can take greater advantage of the SSC. Furthermore, the use of free weight or ground based strength/RT would be highly recommended for youth in order to emphasize initial balance adaptations (Behm et al., 2008, 2010a,b).

The only exception to the strength training advantage for sprint performance was with untrained participants with strength training providing moderate benefits (0.57) compared to large benefits (1.19) with plyometric training. However, upon closer inspection, there were only 3 measures each available for the untrained strength and plyometric training participants vs. 11 and 30 measures for the trained strength and plyometric trained participants, respectively. Hence, with such a sparsity of measures, one must be cautious about interpreting the robustness of this specific result for the untrained youth population.

There are a few youth training studies that combine plyometric and RT. As expected, the combination of plyometrics and RT provided significantly greater improvements in sprint speed and vertical jump height performance than untrained controls with 6 and 12 weeks of training, respectively (Wong et al., 2010; Hopper et al., 2017). Radnor et al. (2017) compared 6 weeks of plyometric, RT and combined training and found more positive responders for 30 m sprint speed with the combined pre-PHV group. In the post-PHV group, the combined training provided more positive responders with acceleration (10 m sprint) and squat jumps vs. the plyometric only and RT groups. Similarly, Kotzamanidis et al. (2005) reported that the combination of 13 weeks of RT and speed training provided greater training benefits for 30 m sprint, squat jump and CMJ than RT alone. The combination of plyometric and RT in these studies did not provide substantially greater training adaptations than the plyometric only training meta-analysis results expressed in this meta-analysis. While Wong et al. (2010) reported small to moderate magnitude improvements for vertical jump height, 10 and 30 m sprint performance, Kotzamanidis et al. (2005) reported 3–7% improvements in sprint and jump performances vs. 1–2% improvements for the RT only group. Thus, the combination of plyometric and strength training exercises did not seem provide additive benefits compared to either plyometric or RT alone.

Untrained youth in this meta-analysis produced greater training gains with jump and sprint measures (for both strength and power training) than trained youth. Table 2 illustrates that not only were the numerical effect sizes greater but in each case the threshold for the magnitude descriptor was exceeded and moved into a higher category with the untrained (i.e., moderate vs. large, small vs. moderate, small vs. large). Since the untrained individuals are beginning a training program and are situated at a lower baseline of functional performance, the initial degree of improvement would be expected to be greater than with trained individuals whose physical capacities have already progressed beyond their initial baseline. Similarly, Behringer et al. (2011) reported a similar trend and offered there might a ceiling effect of functional adaptations in experienced subjects, whereas novices and non-athletes experience greater adaptations due to greater learning effects. The only exception to the untrained groups training accrual benefits was for the effect of strength training upon lower body strength measures, where both groups had large magnitude changes. The training adaptation emphasis may differ between these two groups with untrained youth optimizing motor control/learning and coordination, whereas trained youth may emphasize more the neural (recruitment, rate coding synchronization) and morphological adaptations. So, although the trained youth may be closer to their training potential ceiling, they may be able to tap into adaptations not yet fully available to the untrained.

A limitation of this meta-analysis is that the involved studies investigated relatively healthy and athletic populations. Future studies should also focus on populations with risk factors. Furthermore, appropriate age or maturation matched power and plyometric training intensities, volumes, durations, frequencies and other factors (e.g., What is the optimal platform height for drop jumps with different youth maturational levels? With the appropriate intensity established, what would be the appropriate volume of power training for each session or each week/cycle?) should be investigated to obtain the greatest benefits.

In conclusion, there was modest evidence for the effect of power training specificity upon power measures (small to moderate magnitudes of change). Plausibly due to the greater reaction forces with sprinting, there was no power training specific advantage with sprint results. On the contrary, strength training provided greater sprint training benefits likely due to the development of greater strength allowing the individuals to absorb and react to the ground reaction forces more efficiently to optimize the SSC mechanical and reflexive advantages. Strength training provided the greatest training specific results in youth with consistently large magnitude improvements in lower body strength across trained, vs. untrained, as well as with children vs. adolescents. In addition, untrained youth with their lower baseline of physical capacities (untapped training potentials), immature motor learning (Payne and Isaacs, 2005; Behm et al., 2010b; Behringer et al., 2011; Hopper et al., 2017) and possibly due to their lack of experience tend to experience greater training benefits for power and sprint measures than trained youth. Based on these findings, resistance training for youth should initially emphasize strength training methods. Prior research has also demonstrated the importance of introducing balance training early in the training process (Behm et al., 2008; Hammami et al., 2016b). Plyometric training can also be included but this training should emphasize lower amplitude movements with low to moderate reaction forces (Behm et al., 2008). Proper form, balance and motor control should be first emphasized before presenting the individual with high reaction forces. As indicated in the Canadian Society for Exercise Physiology position stand (Behm et al., 2008), plyometric training and other forms of power training (e.g., Olympic weight lifting) are not intended to be stand-alone exercise programs, the best approach is to incorporate properly supervised and progressive power training into a well-rounded program that also includes other types of strength and conditioning.

## Author contributions

All authors listed have made a substantial, direct and intellectual contribution to the work, and approved it for publication.

### Conflict of interest statement

The authors declare that the research was conducted in the absence of any commercial or financial relationships that could be construed as a potential conflict of interest.
